# Single-cell and spatial multi-omics combined with machine learning to unravel the heterogeneity of cholangiocarcinoma and the WFDC3-mediated prognostic regulatory mechanisms

**DOI:** 10.3389/fimmu.2026.1903609

**Published:** 2026-10-08

**Authors:** Zhencheng Zhu, Tao Jiang, Qiuhua Liu, Bo Zhang, Gang Wang, Pin Huang, Yue Liu, Dingtao Hu, Ke Guo

**Affiliations:** 1 Department of Hepatobiliary Surgery, Zhangjiagang Hospital Affiliated to Soochow University/Zhangjiagang First People’s Hospital, Suzhou, China; 2 Department of Ultrasound, The First Affiliated Hospital of Anhui Medical University, Hefei, China; 3 Department of Oncology, The First Affiliated Hospital of Anhui Medical University, Hefei, China

**Keywords:** cholangiocarcinoma, machine learning, prognostic model, single-cell sequencing, spatial transcriptomics, tumor microenvironment, WFDC3

## Abstract

**Background:**

Intrahepatic cholangiocarcinoma (ICC) exhibits substantial cellular heterogeneity and a complex tumor microenvironment (TME), contributing to poor clinical outcomes. Effective prognostic biomarkers and a deeper understanding of the cellular ecosystems associated with high-risk disease remain urgently needed.

**Methods:**

We integrated five scRNA-seq datasets from GEO with four publicly available spatial transcriptomic sections and bulk transcriptomic data from the TCGA, fu-ICCA, and GEO cohorts. A multidimensional framework combining single-cell analysis, spatial transcriptomics, and machine learning was used to characterize cellular heterogeneity and identify prognostically relevant features. Hotspot was used to characterize T- and B-cell transcriptional modules, Scissor was applied to identify prognosis-associated malignant epithelial cells, and 101 machine-learning pipelines were evaluated to construct the ICCS prognostic signature. Cell–cell communication analysis and exploratory spatial co-density analysis were further performed to characterize ICCS-associated stromal–epithelial interactions, while in silico perturbation and functional assays were used to investigate WFDC3.

**Results:**

The single-cell atlas identified 11 major cell populations and prognosis-associated T- and B-cell transcriptional programs. A six-gene ICCS comprising CEP55, EGLN3, GPRC5A, PRR15, SFN, and WFDC3 showed reproducible prognostic discrimination across the analyzed cohorts. ICCS-High displayed a markedly epithelial-dominant cellular composition, with epithelial cells increasing from 15.47% in ICCS-Low to 78.44% in ICCS-High, whereas T/NK cells decreased from 44.28% to 3.95%. Comparative cell–cell communication analysis revealed prominent mesenchymal-to-epithelial signaling enriched for collagen-related ligand–receptor interactions. COL4A1–CD44 showed the highest relative contribution and a more prominent mesenchymal–epithelial communication pattern in ICCS-High. Exploratory spatial density maps further identified local co-occurrence of mesenchymal and epithelial signals, supporting an ECM-associated microenvironmental context in the high-risk state. Separately, in silico WFDC3 perturbation implicated tumor-related pathways, and WFDC3 knockdown reduced colony formation, migration, and invasion of ICC cells.

**Conclusion:**

ICCS stratification captures distinct cellular and molecular states of the ICC microenvironment. The ICCS-High state is characterized by epithelial predominance, reduced T/NK-cell representation, and stronger inferred mesenchymal-to-epithelial collagen-related communication. COL4A1-CD44 and WFDC3 should be regarded as hypothesis-generating biological features requiring further mechanistic validation. ICCS remains a research-stage prognostic signature that requires standardized assays and independent prospective validation before clinical application.

## Background

Cholangiocarcinoma is the second most common primary hepatic malignancy, second only to hepatocellular carcinoma. Based on its anatomical location within the biliary tree, it can be classified into perihilar cholangiocarcinoma, distal cholangiocarcinoma, and intrahepatic cholangiocarcinoma (ICC). Perihilar cholangiocarcinoma accounts for 50% to 60% of all cases and is defined as a tumor located proximal to the origin of the cystic duct and distal to the secondary bile ducts. Distal cholangiocarcinoma accounts for 20% to 30% of all cases and is located distal to the origin of the cystic duct (1, 2). ICC is an almost universally fatal malignancy, accounting for 20% of all liver malignancies and 3% of all gastrointestinal malignancies (3, 4). The incidence of ICC has increased by more than 140% over the past 40 years (5). For ICC, mortality rates have remained stable in most geographical regions (6–8), with a 5-year overall survival (OS) rate of approximately 9% (9). Major risk factors for ICC include chronic infections, cirrhosis, metabolic factors, biliary tract diseases, environmental toxins, and genetic factors (10). Since 2001, the incidence of cholangiocarcinoma has increased, with a greater increase in the incidence of ICC compared to extrahepatic cholangiocarcinoma.

TME is composed of tumor cells, immune cells, stromal cells, and the extracellular matrix, and its complex cellular interaction network plays a key role in tumor initiation, development, and metastasis (11). Traditional bulk sequencing techniques struggle to capture the heterogeneous characteristics of individual cells, while the development of single-cell RNA sequencing technology has made it possible to accurately analyze the cellular subpopulations and functional states of the TME (12–14). Spatial transcriptomics compensates for the loss of spatial location information in single-cell sequencing, enabling the correlation analysis of gene expression and tissue spatial localization, providing a powerful tool for revealing intercellular spatial interaction mechanisms (15–17). Integrated multi-omics analysis combined with machine-learning approaches can facilitate the identification of prognostically relevant molecular features and the development of candidate prognostic models, thereby providing a framework for further translational investigation (18–20). Previous studies have used single-cell sequencing to analyze the heterogeneity of the immune microenvironment in cholangiocarcinoma, finding that the infiltration of immune cells such as tumor-associated macrophages and regulatory T cells is associated with patient prognosis (12, 17). However, research on the mechanisms of malignant transformation of cholangiocarcinoma epithelial cells, the screening of key prognostic genes, and spatial regulatory networks is still not systematic enough.

This study aimed to integrate single-cell RNA sequencing, spatial transcriptomics, and bulk transcriptomic data to characterize the cellular heterogeneity of ICC, identify prognostically relevant genes, construct a prognostic signature, and investigate the biological role of selected candidate genes, thereby providing a framework for further prognostic and mechanistic studies of ICC.

## Materials and methods

### Data source

Single-cell RNA sequencing (scRNA-seq) data were obtained from the Gene Expression Omnibus (GEO) database, including 5 cholangiocarcinoma-related datasets (GSE125449, GSE138709, GSE142784, GSE171899, GSE181878), covering tumor tissue, adjacent normal tissue, and peripheral blood mononuclear cell (PBMC) samples from cholangiocarcinoma patients, used for single-cell mapping and cell clustering analysis. Spatial transcriptomics data were obtained from a publicly available dataset (http://lifeome.net/supp/livercancer-st/data.htm), including 4 samples (cHC-1N, cHC-1L, cHC-1T, ICC-1L), where cHC represents combined hepatocellular-cholangiocarcinoma, N represents normal tissue, L represents tumor margin tissue, T represents tumor tissue, and ICC represents cholangiocarcinoma tissue, used for spatial localization of cell types and niche analysis. Given the limited number and heterogeneous histological origins of these four spatial sections, all spatial transcriptomic analyses were considered exploratory and hypothesis-generating. They were used to assess spatial localization and concordance with findings from other omics layers, rather than to provide population-level statistical inference or independent clinical validation. Bulk sequencing data from the TCGA cholangiocarcinoma dataset were downloaded from The Cancer Genome Atlas database, including gene expression matrices, clinicopathological parameters, and survival information, used for differential gene screening and functional enrichment analysis. The fu-ICCA dataset was obtained from “Proteogenomic characterization identifies clinically relevant subgroups of intrahepatic cholangiocarcinoma”, containing gene expression data and complete follow-up information for 244 cholangiocarcinoma patients, used for prognostic model training. The merged GEO dataset integrated three datasets (GSE89748, GSE89747, GSE107943), totaling 145 samples, used for external validation of the prognostic model.

### Single-cell data processing and cell clustering

Single-cell RNA-seq data were processed using Seurat (v4.3.1). Cells were retained if they expressed 200–6,000 genes, had a mitochondrial transcript proportion below 20%, and had a ribosomal transcript proportion below 50%. Potential doublets were identified and removed from each sample using DoubletFinder (v2.0.6). DoubletFinder was run with its standard workflow, with pN set to 0.25, pK selected by parameter sweeping based on the maximum BCmetric, and the expected doublet number adjusted according to the estimated homotypic doublet proportion. After quality-control filtering and doublet removal, 82,316 cells were retained for downstream analyses. Gene-expression data were normalized and variance-stabilized using SCTransform. Principal component analysis was performed on the integrated feature space, and dataset-associated variation was mitigated using Harmony with default parameter settings. The Harmony-corrected principal components were subsequently used for nearest-neighbor graph construction, clustering, and Uniform Manifold Approximation and Projection visualization. Cell identities were assigned according to canonical lineage markers, including EPCAM and KRT19 for epithelial cells, CD3D and CD3E for T/NK cells, CD79A and MS4A1 for B cells, LYZ and CD68 for monocytes/macrophages, COL1A1 and ACTA2 for mesenchymal cells, and VWF and CLDN5 for endothelial cells.

### Hotspot module identification and survival analysis

Hotspot (v1.1.3) analysis was performed separately for the CD4+ T-cell, CD8+ T-cell, and B-cell populations using Hotspot. A k-nearest-neighbor graph was constructed in the low-dimensional embedding space using 30 nearest neighbors, with neighborhood_factor set to 3 and weighted_graph set to False. Unless otherwise specified, the default Hotspot settings were used. Gene-wise autocorrelation analysis was performed with the bandwidth parameter set to 0.1. Genes with p< 0.05 were retained for pairwise local-correlation analysis. Gene modules were subsequently identified from the local-correlation matrix using a minimum module size of 20 genes. No manual merging of the automatically identified modules was performed. For patient-level survival analysis, the top 10 representative genes ranked by the Hotspot output were selected from each module. Module activity in each bulk transcriptomic sample was quantified using single-sample gene set enrichment analysis (ssGSEA). Patients were divided into high- and low-module-score groups according to the cohort-specific median ssGSEA score. Overall survival was compared using Kaplan–Meier analysis and the log-rank test. A two-sided p value< 0.05 was considered statistically significant. These module-based survival analyses were regarded as exploratory, and modules were not considered independent prognostic factors unless supported by multivariable Cox regression.

### Epithelial cell CNV analysis

CNV inference was performed using inferCNV (v1.28.0), with mesenchymal used as the reference population. The analysis was performed with a cutoff of 0.1, cluster_by_groups = TRUE, denoise = TRUE, and HMM = TRUE. Cell-level CNV scores were calculated based on the magnitude of inferred CNV alterations across genomic regions. Cells with CNV scores >0.25 were classified as CNV-high and considered putative malignant epithelial cells. To independently validate the inferred CNV status, CopyKAT (v1.2.5) was additionally applied to the epithelial-cell expression matrix using the default parameters. Cells were classified as aneuploid or diploid according to the CopyKAT output, and the resulting classification was compared with the inferCNV-derived CNV scores to assess the consistency of CNV-based malignant-cell identification.

### Spatial niche analysis and key gene screening

Spatial deconvolution was performed using RCTD implemented in spacexr (v2.2.1). The annotated scRNA-seq dataset was used as the reference, with 11 major cell populations serving as reference cell types. RCTD was run separately for each spatial section using doublet_mode = “full” and max_cores = 8. Cell-type weights inferred for each spatial spot were subsequently used for spatial visualization and downstream niche analyses. Seurat software was used to perform clustering analysis on the spatial transcriptomic data to identify spatial niches and screen for differentially expressed genes between different niches. The intersection of differentially expressed genes between tumor and normal tissues from the TCGA dataset, spatial niche differentially expressed genes, and Scissor+ genes was taken to obtain a set of key genes (65 genes). Because of the limited spatial sample size, niche-related differential expression was analyzed at the section level and was used only as one exploratory candidate-prioritization layer. No population-level generalization was inferred from the spatial transcriptomic data alone. Candidate genes identified through the spatial analysis were subsequently evaluated using independent bulk transcriptomic and survival cohorts.

### Prognostic model construction and validation

One hundred and one machine learning algorithms were used to screen 65 key genes for prognostic gene selection and model construction. The optimal model was selected using the concordance index (C-index) and the area under the receiver operating characteristic (ROC) curve (AUC) as evaluation metrics. Six prognostic genes were finally selected through LASSO regression, to quantify the prognostic risk of each patient, a multigene risk score model ICCS was constructed based on the expression levels of six selected genes (SFN, EGLN3, GPRC5A, CEP55, PRR15, and WFDC3). The final ICCS score was calculated using the following fixed formula: ICCS = (0.113 × SFN expression) + (0.203 × EGLN3 expression) + (0.078 × GPRC5A expression) + (0.424 × CEP55 expression) + (0.091 × PRR15 expression) + (0.052 × WFDC3 expression). And the prognostic model ICCS was constructed with the formula:


$$\text{RiskScore}=\sum_{\text{i}=1}^{\text{n}}\left({\text{Coef}}_{\text{i}}\times {\text{Exp}}_{\text{i}}\right)$$

where Coef_i_ is the coefficient of gene *i* derived from the multivariate Cox regression analysis, and Exp_i_ is the expression value of gene *i*. The prognostic performance of ICCS was evaluated in the fu-ICCA and GEO merged datasets using Kaplan–Meier survival analysis, time-dependent ROC analysis, and Cox regression. Univariable and multivariable Cox regression analyses were used to assess the associations of ICCS and the individual genes with overall survival after adjustment for the available clinical variables. This median-based threshold was used for analytical stratification and should not be interpreted as a clinically validated cutoff.

### Comparison with published prognostic signatures

To benchmark ICCS against existing ICC prognostic signatures, previously published models with available gene lists and clearly reported scoring procedures were reconstructed using the same transcriptomic cohorts. For each signature, risk scores were calculated according to the scoring method reported in the original publication. Only genes available in the corresponding evaluation dataset were included, and models lacking sufficient gene coverage or reproducible scoring information were excluded.The prognostic discrimination of ICCS and the published signatures was evaluated using the concordance index in the fu-ICCA, test, and MetaCohort datasets. Confidence intervals were calculated for the C-index estimates. Additional associations of ICCS with overall survival and disease-free survival were evaluated in GSE107943 using univariable Cox regression. Kaplan–Meier analyses were performed after stratifying patients according to the median ssGSEA score of the six-gene ICCS signature.

### Immune infiltration analysis

The CIBERSORT algorithm was used to assess the immune cell infiltration levels of samples in different risk groups (high/low ICCS score groups), and differentially infiltrated immune cell subpopulations were screened. Single-sample gene set enrichment analysis (ssGSEA) was used to assess the activity differences of immune-related pathways. The association between differential immune cell infiltration and patient prognosis was analyzed using the Kaplan-Meier method.

### Key gene functional validation

Virtual knockout analysis was performed using scTenifoldKnk (v1.0.2) on malignant epithelial cells. WFDC3 was specified as the target gene, with n_pcs = 50, n_neighbors = 15, and n_epochs = 100. Genes showing an absolute fold change (|FC|) >1 were retained for downstream functional enrichment analysis. Gene Ontology (GO) and Kyoto Encyclopedia of Genes and Genomes (KEGG) pathway enrichment analyses were performed using clusterProfiler (v4.13.4), with an adjusted P-value<0.05 considered statistically significant.

### Cell communication analysis

Tumor-derived single cells were stratified into ICCS-Low and ICCS-High states according to the predefined ICCS scoring strategy. Intercellular communication among the 11 major cell populations was inferred using CellChat (v2.2.0) following the standard workflow for identifying overexpressed signaling genes and ligand–receptor interactions, computing communication probabilities, and aggregating cell-type-level interaction strengths (21). Overall interaction patterns were compared between ICCS-Low and ICCS-High states. To specifically characterize stromal–epithelial communication, mesenchymal cells were designated as source cells and epithelial cells as target cells. Significant ligand–receptor interactions were retained using the statistical threshold applied in the analysis, and collagen-related interactions involving CD44, syndecans, and integrin receptor complexes were summarized. The relative contribution of each ligand–receptor pair to mesenchymal-to-epithelial signaling was subsequently quantified. Because COL4A1–CD44 showed the highest relative contribution, its cell-type-level communication network was visualized separately in ICCS-Low and ICCS-High states. For exploratory spatial validation, mesenchymal and epithelial cell signals were mapped across the cHC-1T, cHC-1L, and ICC-1L spatial transcriptomic sections, and local regions showing co-occurrence of mesenchymal and epithelial signals were visualized.

### qRT-PCR

Total RNA extraction utilized TRIzol reagent (Invitrogen, Carlsbad, California, USA) following the manufacturer’s instructions. The concentration and purity of the extracted RNA were assessed using the NanoDrop 2000 spectrophotometer (Thermo Fisher Scientific, USA). Subsequently, 1 μg of total RNA underwent reverse transcription into cDNA with PrimeScript RT Master Mix (Takara Bio, Shiga, Japan). Quantitative real-time PCR (qRT-PCR) was performed using SYBR Green PCR Master Mix (Applied Biosystems, Foster City, California, USA) in the QuantStudio 7 Flex real-time PCR system (Thermo Fisher Scientific). The specific primer sequences (5’-3’) are as follows: GAPDH (forward: GGTGAAGGTCGGTGTGAACG, reverse: CTCGCTCCTGGAAGATGGTG), CEP55 (forward: TCTCTTCGATCGCTTCTTTGG, reverse: GGCTTCGATCCCCACTTACT), EGLN3 (forward: ATCGACAGGCTGGTCCTCTA, reverse: AGCCACCATTGCCTTAGACC), GPRC5A (forward: CTTTCCCAGGAGGTGTGACC, reverse: CCCATGCCTGGATCCTGTTT), PRR15 (forward: GTCCTGTTAAGCCCGAGCAG, reverse: TCTGCTGTTGGTGAGCGATT), SFN (forward: GAGCGAAACCTGCTCTCAGT, reverse: ATGACCAGTGGTTAGGTGCG), and WFDC3 (forward: GTTGGGGCTTCCTCTCGGG, reverse: GTCCAGTTGGGGCATGGT). qRT-PCR measurements were performed using three independent biological replicates per group. For each biological replicate, qPCR reactions were run in three technical replicate wells, and the mean Ct value of the technical replicates was used for downstream calculation.

### WFDC3 knockdown and functional assays

ICC cells were transfected with either a negative-control shRNA (NC) or an shRNA targeting WFDC3 (sh-WFDC3). Total cellular protein was extracted from independently cultured cell preparations, separated by SDS-PAGE, and transferred to membranes for Western blot analysis. WFDC3 and GAPDH were detected by immunoblotting, and WFDC3 protein expression was normalized to GAPDH to assess knockdown efficiency. Western blot experiments were independently repeated three times, with separately prepared cell lysates representing three biological replicates.

For functional validation, NC and sh-WFDC3 cells were subjected to colony formation, wound-healing, and Transwell invasion assays. All functional assays were performed in three independent biological experiments. For the colony formation assay, cells were seeded at low density in three replicate wells per condition within each biological experiment, cultured until visible colonies formed, and then fixed, stained, photographed, and counted. Colony numbers from the replicate wells were averaged to obtain one value for each biological replicate.

For the wound-healing assay, confluent cell monolayers were scratched with a sterile pipette tip, and images were acquired at 0 and 24 h. Three replicate wells were analyzed for each condition in each independent experiment. Wound-healing rates were calculated for each well, and the replicate-well measurements were averaged to generate one value per biological replicate.

For the Transwell invasion assay, cells were seeded into three replicate Transwell inserts per condition in each independent experiment. After incubation, cells that had invaded through the membrane were fixed and stained. Cells were quantified from representative non-overlapping microscopic fields in each insert, and field-level measurements were averaged for each insert before calculation of the biological-replicate value. Technical replicates within the same independent experiment were averaged and were not treated as independent biological replicates for statistical analysis. Unless otherwise specified, quantitative data were derived from three independent biological replicates.

### Software versions and computational reproducibility

All computational analyses were performed using R (v4.3.3) and Python (v 3.12.12). The principal software packages included Seurat (v4.3.1), DoubletFinder (v2.0.6), Harmony (v2.0.5, Hotspot (v1.1.3), inferCNV (v1.28.0), Scissor (v2.1.0), spacexr (v2.2.1), scTenifoldKnk (v1.0.2), CellChat (v2.2.0), and clusterProfiler (v4.13.4). The exact package versions, software environments, input data, and complete analysis parameters are additionally summarized in **Supplementary Table S1** and the accompanying reproducibility repository.

### Statistical analysis

All statistical analyses were performed using R software (v4.3.3). Measurement data were expressed as mean ± standard deviation, and t-tests or ANOVA were used for group comparisons; count data were expressed as frequencies and percentages, and χ² tests were used for group comparisons. Survival analysis was performed using the Kaplan-Meier method and the log-rank test. A *P*-value< 0.05 was considered statistically significant. Detailed information on the software versions, input data, key parameter settings, and validation or robustness procedures used in the principal computational analyses is provided in **Supplementary Table S1**.

## Results

### Single-cell atlas construction and cell clustering of cholangiocarcinoma

To characterize the heterogeneous immune microenvironment of ICC, we integrated five GEO scRNA-seq datasets (GSE125449, GSE138709, GSE142784, GSE171899, and GSE181878) using Harmony after quality-control filtering. Harmony-corrected UMAP visualization showed the distribution of cells according to GEO dataset source (**Supplementary Figure S1c**), while quantitative integration metrics demonstrated improved dataset mixing after Harmony correction (**Supplementary Figure S1d**). Quantitatively, the scaled iLISI score increased from 0.000 to 0.082 and the cell-type-aware kBET acceptance rate increased from 0.018 to 0.366, whereas the scaled cLISI score remained at 1.000 (**Supplementary Figure S1d**). These results indicated improved dataset mixing with preservation of the major cell-type structure, although residual dataset-associated effects remained. Harmony-corrected UMAPs further showed cell distributions by individual sample and disease group (**Supplementary Figures S1a, b**).

The integrated cells were classified into 11 major cell types: B cells, endothelial cells, epithelial cells, hepatoblasts, IRF7+ pDC cells, mesenchymal cells, monocytes/macrophages, myeloid cells, plasma cells, Schwann cells, and T/NK cells (**Figures 1A–C**). Cell identities were confirmed using canonical markers, including CD79A/MS4A1 for B cells, VWF/CLDN5 for endothelial cells, EPCAM/KRT19 for epithelial cells, ALB/APOA2 for hepatoblasts, IRF7/LILRA4 for pDCs, COL1A1/ACTA2 for mesenchymal cells, CD14/CD68 for monocytes/macrophages, LYZ/TYROBP for myeloid cells, MZB1/XBP1 for plasma cells, S100B/CPVL for Schwann cells, and CD3D/NKG7 for T/NK cells (**Figures 1D, E**).

**Figure 1:**
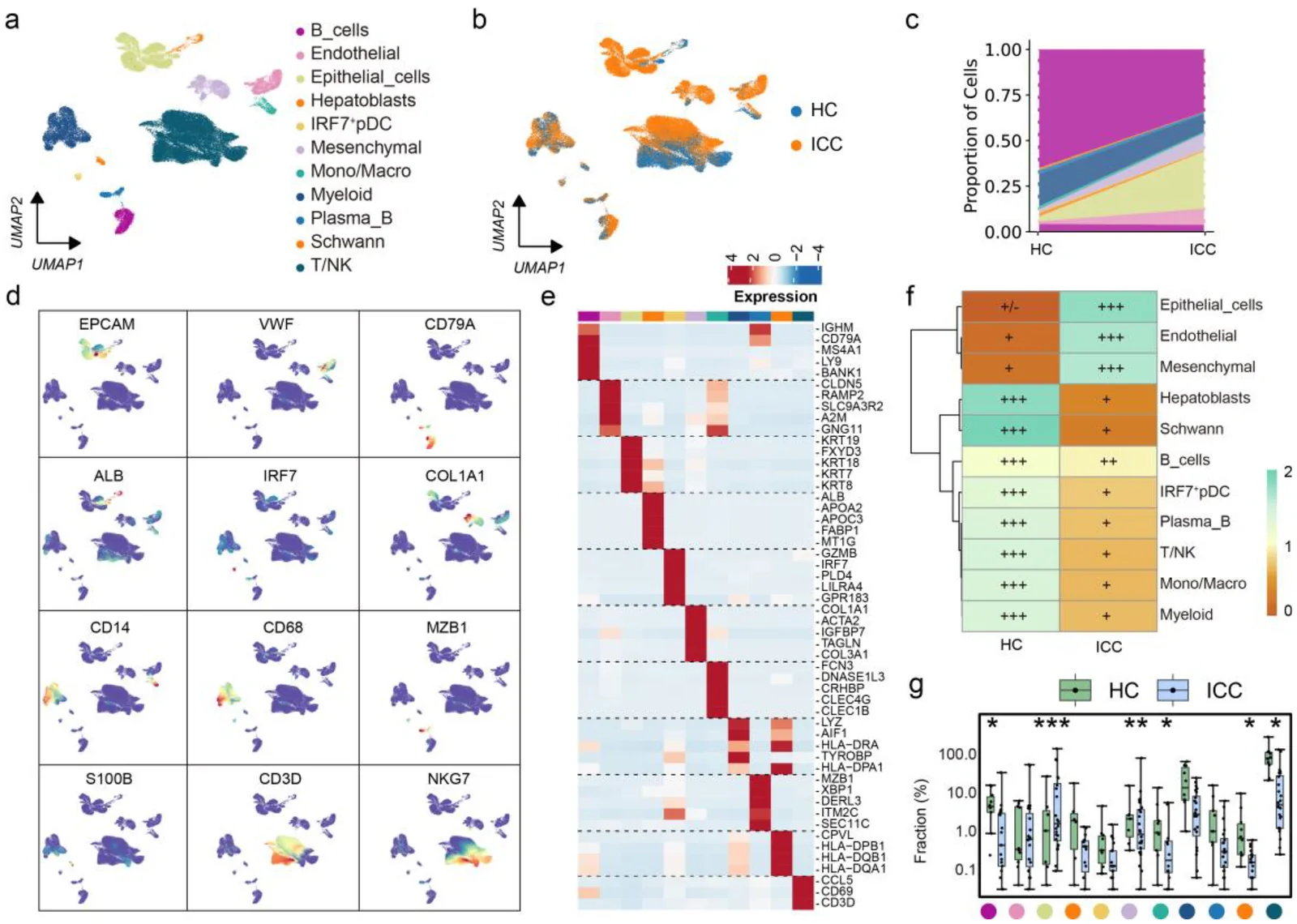
Determination of the major cellular compositions of TME in the single-cell atlas of ICC. **(A)** Integration of five ICC single-cell RNA sequencing datasets (GSE125449, GSE138709, GSE142784, GSE171899, and GSE181878) using the Harmony algorithm, with UMAP dimensionality reduction visualization revealing the major cell types (intrahepatic cholangiocarcinoma group, ICC); **(B)** UMAP dimensionality reduction visualization revealing the patient labels; **(C)** The proportional representation of eleven major cell types across the HC and ICC groups; **(D)** The top 5 marker genes of every single major cell type in UMAP; **(E)** The top marker genes of every single major cell type in a heatmap; **(F)** Tissue preference analysis (Ro/e algorithm) revealing proliferating cells in ICC as well as the relative abundance of B cells, endothelial, epithelial cells, hepatoblasts, IRF7^+^pDC, mesenchymal, Mono/Macro, myeloid, Plasma_B, Schwann, and T/NK cells. **(G)** Comparison of major cell type proportions between HC and ICC patient groups. Statistical significance is indicated as follows: *P< 0.05, **P< 0.01, ***P< 0.001.

Ro/e analysis revealed preferential enrichment of epithelial, endothelial, and mesenchymal cells in ICC samples (**Figure 1F**), and epithelial cells were more abundant in ICC than in HC samples (**Figure 1G**). Pathway analysis further identified cell-type-specific immune, metabolic, signaling, and proliferation programs (**Supplementary Figure S1e**), while the sample-level proportion plot showed marked inter-sample variation in cell composition (**Supplementary Figure S1f**).

### T-cell heterogeneity, differentiation trajectories, and prognostic modules

Re-clustering identified seven T-cell subpopulations: CD4+ effector T cells (CD4_Tef_CCL5), CD4+ memory T cells (CD4_Tmem_CD69), CD4+ naive T cells (CD4_Tna_CCR7), regulatory T cells (CD4_Tre_FOXP3), CD8+ effector T cells (CD8_Tef_GZMA), CD8+ memory T cells (CD8_Tmem_LTB), and proliferating T cells (Tpr_TOP2A) (**Figure 2A**). Supplementary feature plots further confirmed CD3D and CD8A expression in the re-clustered T-cell compartment, while the stacked composition plot and additional Hotspot module maps illustrated group-level compositional differences and heterogeneous distributions of the remaining CD8+ T-cell transcriptional modules (**Supplementary Figure S2a**-**d**). Their identities were supported by representative markers, including PRF1 and CCL5 for effector T cells, CCR7 and SELL for naïve T cells, and FOXP3 and IL2RA for regulatory T cells, as further summarized by dot-plot analysis across all seven subpopulations (**Figure 2B**; **Supplementary Figure S2c**).Exploratory spatial mapping showed distinct distributions of T-cell subpopulations across the four tissue sections (**Figure 2C**). Ro/e and proportion analyses revealed marked differences in T-cell composition between HC and ICC samples. ICC samples showed an increased proportion of regulatory CD4+ T cells and reduced proportions of effector CD4+ T cells and memory CD8+ T cells, whereas the reduction in effector CD8+ T cells did not reach statistical significance (**Figures 2D, E**). Hotspot analysis of CD8+ T cells identified eight transcriptional modules (M1–M8) (**Figure 2F**; **Supplementary Figure S2c**), with the complete gene-level Hotspot statistics and module assignments provided in **Supplementary Table S3**. High M1 expression was associated with longer overall survival, whereas high M7 expression was associated with shorter overall survival (**Figures 2G, H**), indicating that selected CD8+ T-cell transcriptional programs were associated with prognosis.

**Figure 2:**
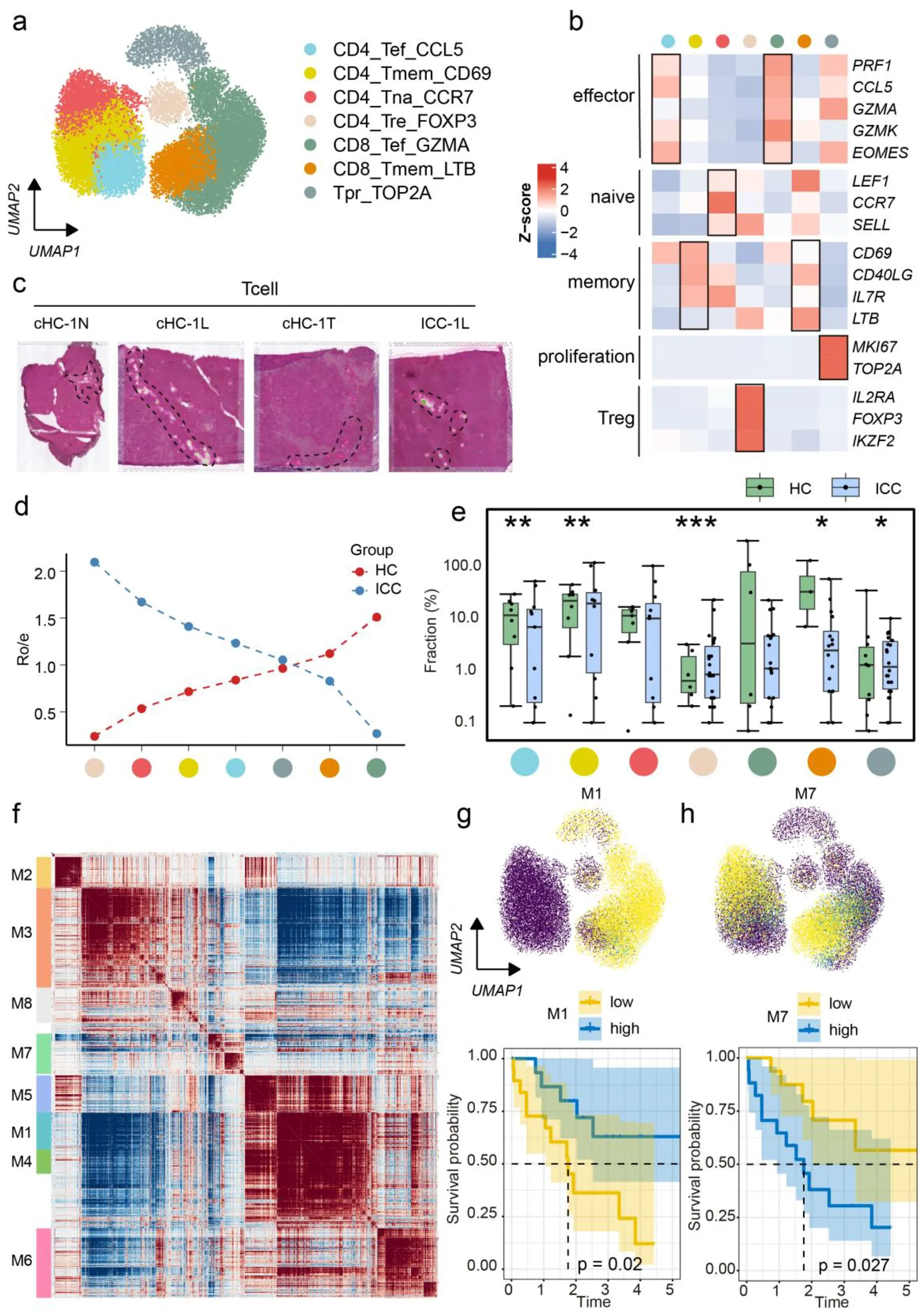
T-cell heterogeneity and prognostic analysis of CD8+ T-cell Hotspot modules. **(A)** UMAP visualization of seven T-cell subpopulations. **(B)** Heatmap showing representative marker-gene expression across the T-cell subpopulations. **(C)** Exploratory spatial mapping of T cells in cHC-1N, cHC-1L, cHC-1T, and ICC-1L sections. **(D)** Ro/e scores showing the distribution preference of T-cell subpopulations in HC and ICC samples. **(E)** Comparison of T-cell subpopulation proportions between the HC and ICC groups. **(F)** Heatmap of eight CD8+ T-cell Hotspot modules (M1–M8). **(G, H)** For the survival analyses in panels **(G, H)**, patients were stratified into high- and low-module-score groups using the cohort-specific median score. Statistical significance is indicated as follows: *P< 0.05, **P< 0.01, ***P< 0.001.

CD4+ T-cell trajectory analysis revealed a branched differentiation pattern originating mainly from CD4_Tna_CCR7 cells and extending toward memory, effector, and regulatory states (**Supplementary Figures S3A–C**). ICC-derived CD4+ T cells tended to occupy later pseudotime states, while functional enrichment indicated that the differentiation branches were associated with distinct metabolic and stress-response programs (**Supplementary Figure S3d**). Hotspot analysis further identified nine CD4+ T-cell transcriptional modules (M1–M9), with the complete gene-level Hotspot statistics and module assignments provided in **Supplementary Table S2**. Among these modules, M2 and M7 were significantly associated with overall survival (**Supplementary Figures S3e–g**).

### B-cell subpopulation heterogeneity, inferred state-transition patterns, and prognostic associations

UMAP clustering partitioned ICC B cells into five subpopulations: naive B cells (B_naive_IGHM), two plasma-cell subpopulations (B_plasma_MZB1 and B_plasma_IGHG1), memory B cells (B_mem_GPR183), and proliferating B cells (B_cycling_TOP2A) (**Figure 3A**). Marker-expression analysis supported these annotations: naive B cells expressed IGHM, plasma cells expressed IGHG1 and MZB1, memory B cells expressed GPR183, and proliferating B cells expressed TOP2A (**Figure 3B**).The proportion of B_plasma_IGHG1 cells was higher in ICC samples than in HC samples, although the difference did not reach statistical significance (**Figure 3C**). Therefore, this observation was interpreted as a descriptive change in B-cell composition rather than evidence of enhanced plasma-cell differentiation. RCTD-based exploratory spatial mapping showed distinct distributions of the B-cell populations across the analyzed tissue sections and an overall reduction in the B-cell signal in the ICC section (**Supplementary Figure S4a**).

**Figure 3:**
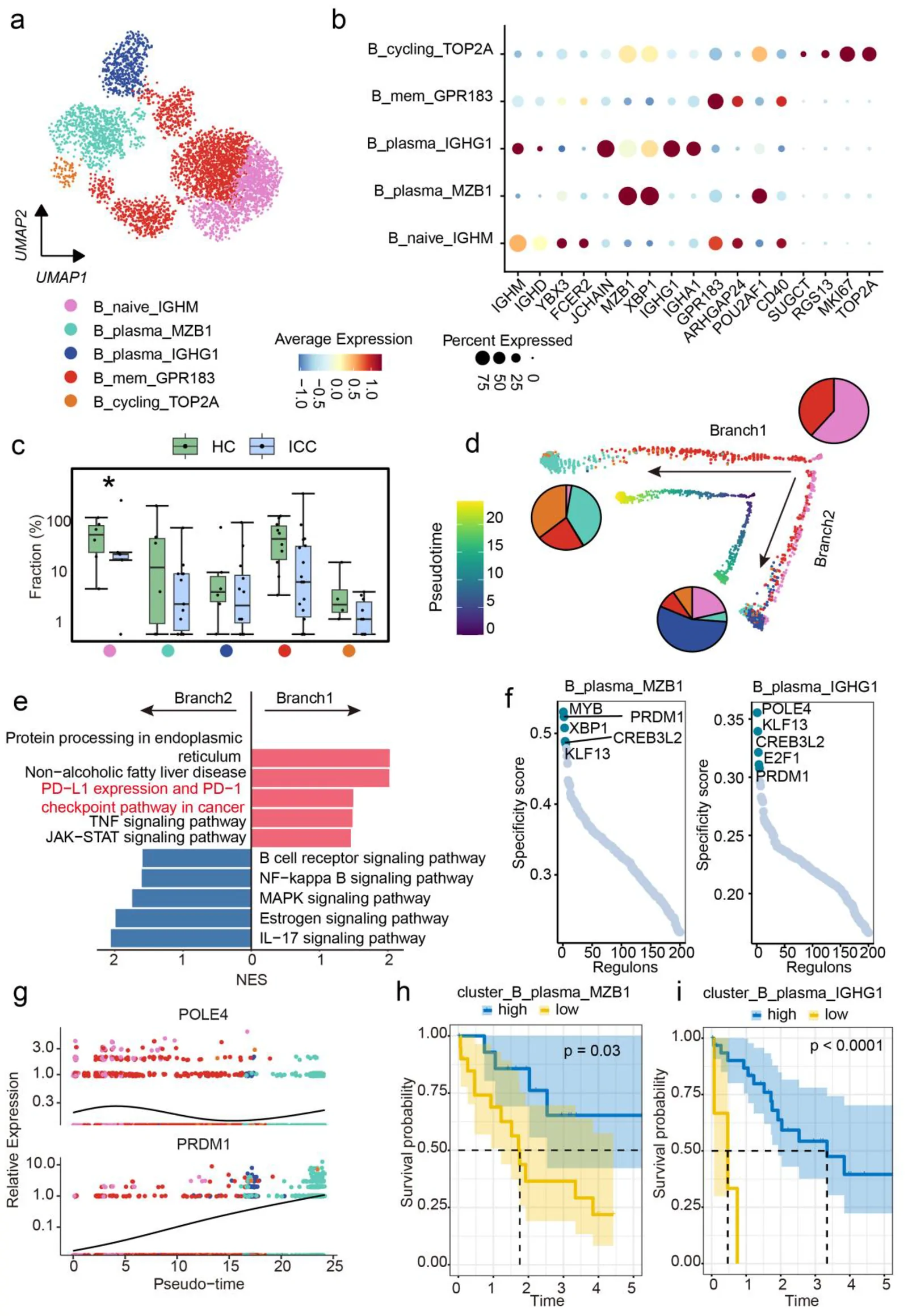
B cell subgroup heterogeneity, differentiation trajectory, and prognostic association. **(A)** UMAP dimensionality reduction clustering map of B cell subgroups, with different colors representing 5 functional subgroups; **(B)** Expression map of core markers of B cell subgroups; **(C)** Bar chart showing the differences in the proportion of each B cell subgroup in HC and ICC samples; **(D)** Inferred state-transition trajectory of B cells along the pseudotime axis, distinguishing two putative branches: Branch 1 and Branch 2; **(E)** Heatmap of functional pathway enrichment in Branch 1 and Branch 2 branches; **(F)** Specificity score curve of core regulatory factors in plasma cell subgroups; **(G)** Expression dynamics of POLE4 and PRDM1 during pseudotime progression; **(H, I)** Kaplan-Meier survival curves of high and low expression groups of B_plasma_MZB1 and B_plasma_IGHG1 subgroups (*p* = 0.03, *p<* 0.0001). Statistical significance is indicated as follows: *P< 0.05.

Pseudotime analysis inferred two putative state-transition branches, designated Branch 1 and Branch 2. Naive B cells were located near the inferred starting region, whereas plasma-cell populations were mainly distributed toward Branch 1 (**Figure 3D**). Functional enrichment suggested that Branch 1 was associated with protein processing in the endoplasmic reticulum, the PD-L1/PD-1 checkpoint pathway, and TNF signaling, whereas Branch 2 was associated with B-cell receptor and NF-κB signaling (**Figure 3E**). The complete pathway-enrichment results for the two inferred B-cell trajectory branches are provided in **Supplementary Table S4**. These patterns represent computationally inferred transcriptional-state relationships rather than experimentally validated developmental lineages. Specificity-score analysis identified MYB, PRDM1, XBP, CREB3L2, and KLF13 as candidate regulators of the B_plasma_MZB1 population, whereas POLE4, KLF13, CREB3L2, E2F1, and PRDM1 were identified as candidate regulators of the B_plasma_IGHG1 population (**Figure 3F**). Along the inferred pseudotime axis, POLE4 expression initially decreased and subsequently increased, whereas PRDM1 expression progressively increased (**Figure 3G**). These expression patterns were associated with the inferred plasma-cell state transition but did not establish direct regulatory effects. Hotspot analysis identified nine B-cell transcriptional modules (M1–M9), which were annotated according to their representative genes and expression patterns (**Supplementary Figures S4b, c**), with the complete gene-level Hotspot statistics and module assignments provided in **Supplementary Table S5**. For survival analysis, ssGSEA scores were calculated using the top 10 representative genes of the corresponding B-cell signatures. Lower B_plasma_MZB1 and B_plasma_IGHG1 signature scores were associated with shorter overall survival (**Figures 3H, I**), suggesting that reduced activity of these plasma-cell-associated transcriptional programs was associated with poorer prognosis.

### Malignant epithelial cell identification and key gene screening

To dissect malignant heterogeneity and spatial functional traits of ICC epithelial cells, we performed fine-grained clustering of tumor epithelial cells. UMAP analysis identified 10 epithelial subpopulations, designated Epi_0 to Epi_9 (**Figure 4A**). InferCNV analysis, using normal epithelial cells as the reference population, revealed broad chromosome-scale copy-number variation and substantial inter-cluster heterogeneity among the epithelial cells (**Figures 4B–D**). To independently evaluate the inferred CNV states, we further applied CopyKAT to 13,506 epithelial cells. CopyKAT classified the cells into aneuploid and diploid populations and showed their distribution across the epithelial-cell UMAP (**Supplementary Figures S5a, b**). CopyKAT-derived scores showed a weak but statistically significant positive correlation with the InferCNV-derived CNV scores (**Supplementary Figure S5c**). The CNV score distributions of CopyKAT-defined aneuploid and diploid cells showed substantial overlap (**Supplementary Figure S5d**), indicating partial rather than complete agreement between the two algorithms. Therefore, these CNV-supported epithelial populations were interpreted as putative malignant epithelial cells.

**Figure 4:**
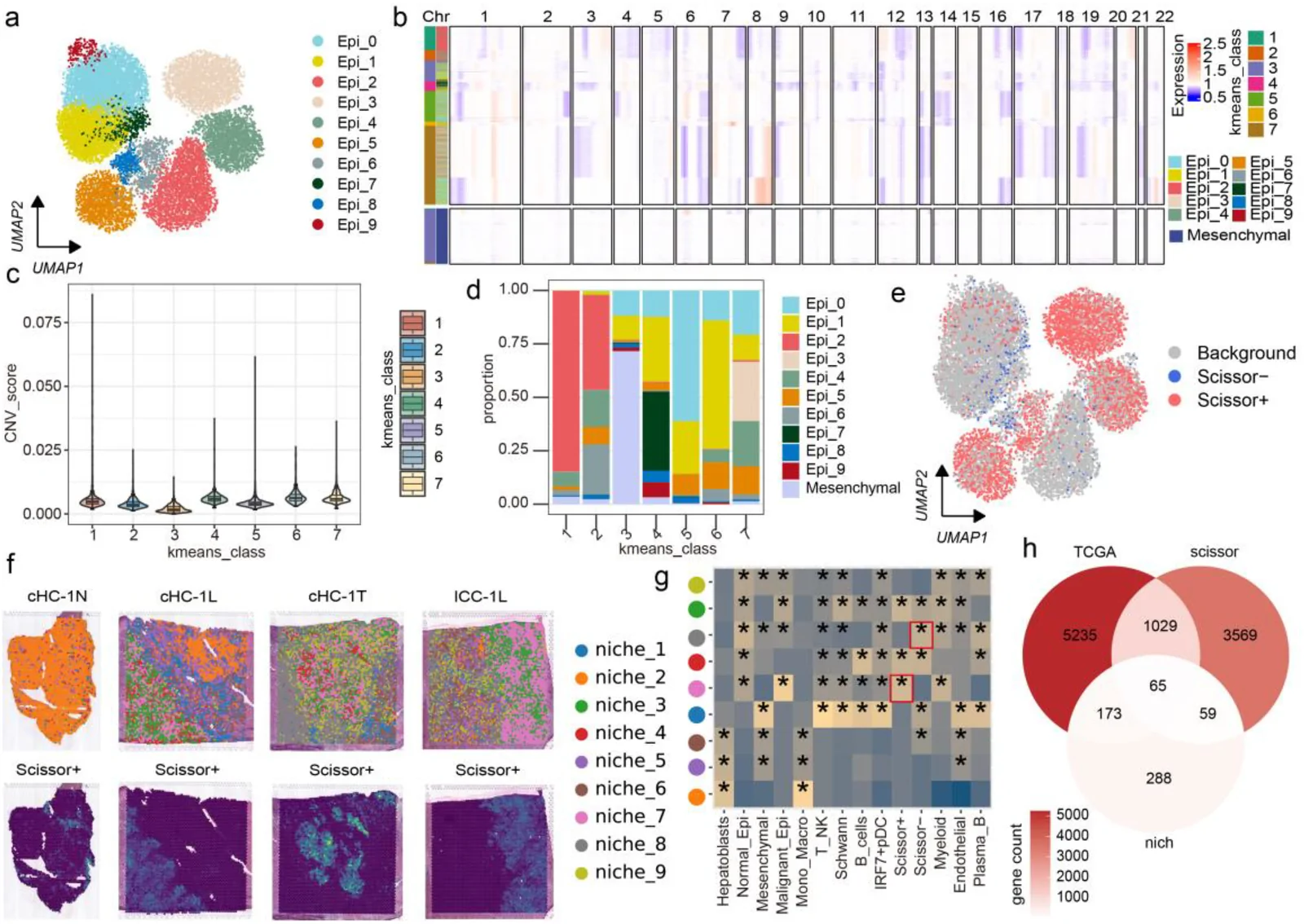
Identification, spatial localization, and key functional gene screening of malignant epithelial cell subpopulations. **(A)** UMAP clustering of tumor epithelial cells, identifying 10 subpopulations (Epi_0 to Epi_9); **(B)** Whole-genome CNV map obtained from InferCNV analysis, with kmeans clustering (1–7) showing different CNV patterns; **(C)** Box plot of CNV scores for each kmeans cluster; **(D)** Stacked bar chart of cell proportions for each kmeans cluster; **(E)** Scissor analysis of putative malignant epithelial cells, distinguishing Scissor^+^ and Scissor^−^ populations according to their association with the survival phenotype. **(F)** Using RCTD deconvolution results for spatial niche analysis (top), and mapping Scissor^+^ subpopulations onto spatial transcriptomics for visualization (bottom), showing their enrichment in tumor regions of cHC and ICC samples. **(G)** Spatial niche partitioning and niche distribution of Scissor subpopulations; **(H)** Venn diagram intersection analysis of three sets of differentially expressed genes, yielding 65 core genes for subsequent machine learning modeling. Statistical significance is indicated as follows: *P< 0.05.

Using bulk RNA-seq survival data as the phenotype input, Scissor further classified the putative malignant epithelial cells into Scissor+ and Scissor− populations (**Figure 4E**). RCTD-based spatial mapping showed preferential localization of Scissor+ cells in tumor-associated regions of the analyzed cHC and ICC sections (**Figure 4F**). The corresponding spot-level cell-type and Scissor-state weights across the four spatial transcriptomic sections are provided in **Supplementary Table S7**. The supplementary spatial maps further demonstrated pronounced section-specific heterogeneity in the distribution of major cell populations across the analyzed cHC and ICC tissue sections (**Supplementary Figure S6**). Spatial niche partitioning identified nine niches, designated niche_1 to niche_9, with the spot-level niche assignments across the four spatial transcriptomic sections provided in **Supplementary Table S6**. Scissor+ cells were mainly distributed in niche_7, whereas Scissor− cells were more frequently observed in niche_8 (**Figure 4G**), suggesting section-level spatial preferences of the two Scissor-defined populations. Finally, we intersected three gene sets, genes differentially expressed between ICC and HC in the TCGA cohort, genes differentially expressed between Scissor+ and Scissor− cells, and genes differentially expressed between niche_7 and niche_8. This analysis identified 65 shared candidate genes (**Figure 4H**), which were subsequently used for feature selection and prognostic model construction.

### Establishment and comparative evaluation of a six-gene prognostic model

Using the 65 candidate genes identified in **Figure 4**, we evaluated 101 machine-learning pipelines across the available fu-ICCA and GEO cohorts. Among these pipelines, Lasso + plsRcox yielded the highest observed C-index, with values of 0.780 in the fu-ICCA cohort and 0.649 in the combined GEO cohort (**Figure 5A**). Subsequent Lasso regression retained six genes—CEP55, EGLN3, GPRC5A, PRR15, SFN, and WFDC3—at an optimal lambda value of 0.0351 (**Figure 5B**). The resulting ICCS was calculated as follows, ICCS = (0.113 × SFN expression) + (0.203 × EGLN3 expression) + (0.078 × GPRC5A expression) + (0.424 × CEP55 expression) + (0.091 × PRR15 expression) + (0.052 × WFDC3 expression).Patients were stratified into high- and low-score groups using the cohort-specific median ICCS. High ICCS was associated with shorter overall survival in both the fu-ICCA and combined GEO cohorts (**Figures 5C, D**). In the fu-ICCA cohort, the time-dependent AUCs at 1, 2, and 3 years were 0.788, 0.782, and 0.771, respectively. The corresponding AUCs in the combined GEO cohort were 0.674, 0.667, and 0.719, respectively (**Figures 5E, F**).

**Figure 5:**
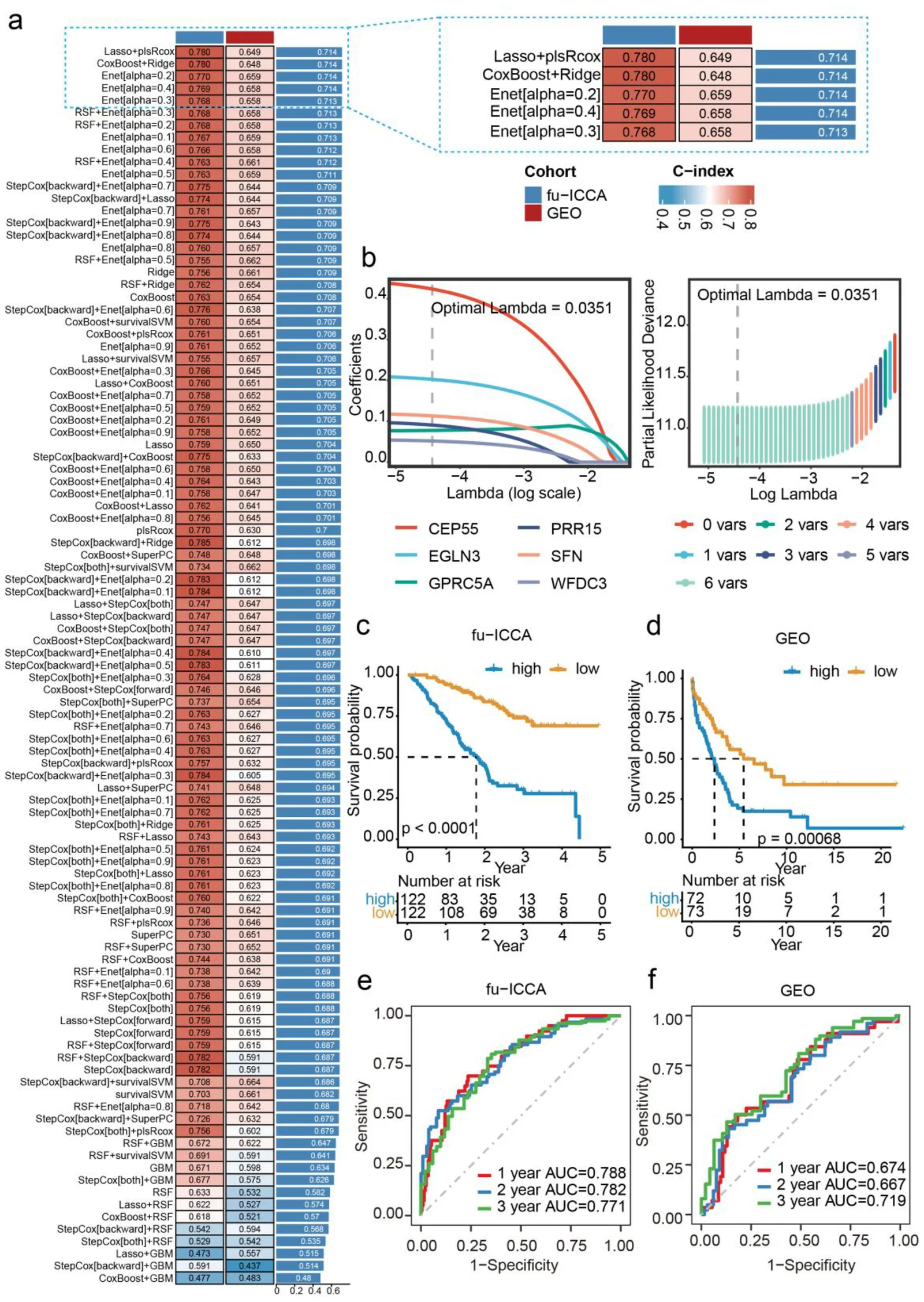
Construction and cross-cohort prognostic evaluation of the six-gene ICCS model. **(A)** Heatmap comparing the C-index of 101 machine learning algorithms in the combined cohort (fu-ICCA + GEO), showing that the Lasso + plsRcox algorithm performed best. **(B)** Coefficient trajectory plot (left) and partial likelihood deviance plot (right) of Lasso regression analysis, determining the optimal Lambda value as 0.0351 and identifying 6 key prognostic genes; **(C, D)** The fu-ICCA cohort included 244 patients, and the combined GEO cohort included 145 samples. Patients were stratified into ICCS-High and ICCS-Low groups using the cohort-specific median ICCS score. Kaplan-Meier survival curves for high and low-risk groups in the fu-ICCA cohort **(C)** and GEO cohort **(D)**; **(E, F)** Time-dependent ROC curves for the fu-ICCA cohort **(E)** and GEO cohort **(F)**, demonstrating the prognostic predictive performance (AUC values) of the model at 1, 2, and 3 years.

To benchmark ICCS against previously published ICC prognostic signatures, we reconstructed the published models using their reported gene sets and scoring methods and evaluated them in the same fu-ICCA, test, and MetaCohort datasets. ICCS achieved the highest C-index in the fu-ICCA cohort and remained among the top-performing signatures in the test cohort and MetaCohort, with performance comparable to the Fan_OSRG and Ji signatures (**Supplementary Figure S7a**). Thus, ICCS showed competitive cross-cohort prognostic discrimination, although it was not consistently superior to all published models. In GSE107943, higher ICCS values were associated with poorer disease-free survival and overall survival (**Supplementary Figure S7b**). Consistently, high ssGSEA scores for the six-gene signature were associated with shorter overall survival and disease-free survival (**Supplementary Figures S7c–d**). Given the wide confidence intervals in the ssGSEA-based analyses, these findings were interpreted as exploratory evidence of prognostic reproducibility.

### Cross-cohort evaluation of ICCS and nomogram construction

We first examined the expression patterns of the six ICCS genes in TCGA-CHOL and GSE107943. CEP55, EGLN3, GPRC5A, PRR15, SFN, and WFDC3 showed higher expression in tumor samples than in normal controls in both cohorts (**Figures 6A, B**). Paired tumor-versus-adjacent-normal analyses in TCGA-CHOL and GSE107943 showed consistent expression differences, further supporting the reproducibility of the tumor-associated expression patterns of the six genes (**Figures 6C, D**).We next evaluated the prognostic associations of the individual ICCS genes and the composite ICCS score. In the fu-ICCA cohort, all six genes and ICCS were associated with overall survival in univariable Cox regression (**Figure 6E**). Among these variables, ICCS showed the strongest observed association with shorter overall survival. After adjustment for the available clinical covariates of age and sex, ICCS remained significantly associated with shorter overall survival (**Figure 6F**). Consistent findings were observed in the merged GEO cohort, where ICCS was associated with shorter overall survival in univariable analysis (**Figure 6G**) and remained prognostically informative after adjustment for age and sex (**Figure 6H**). These results support the reproducibility of the prognostic association of ICCS across the analyzed cohorts, although residual confounding cannot be excluded because several clinically relevant clinicopathological variables were unavailable or could not be consistently incorporated across datasets.

**Figure 6:**
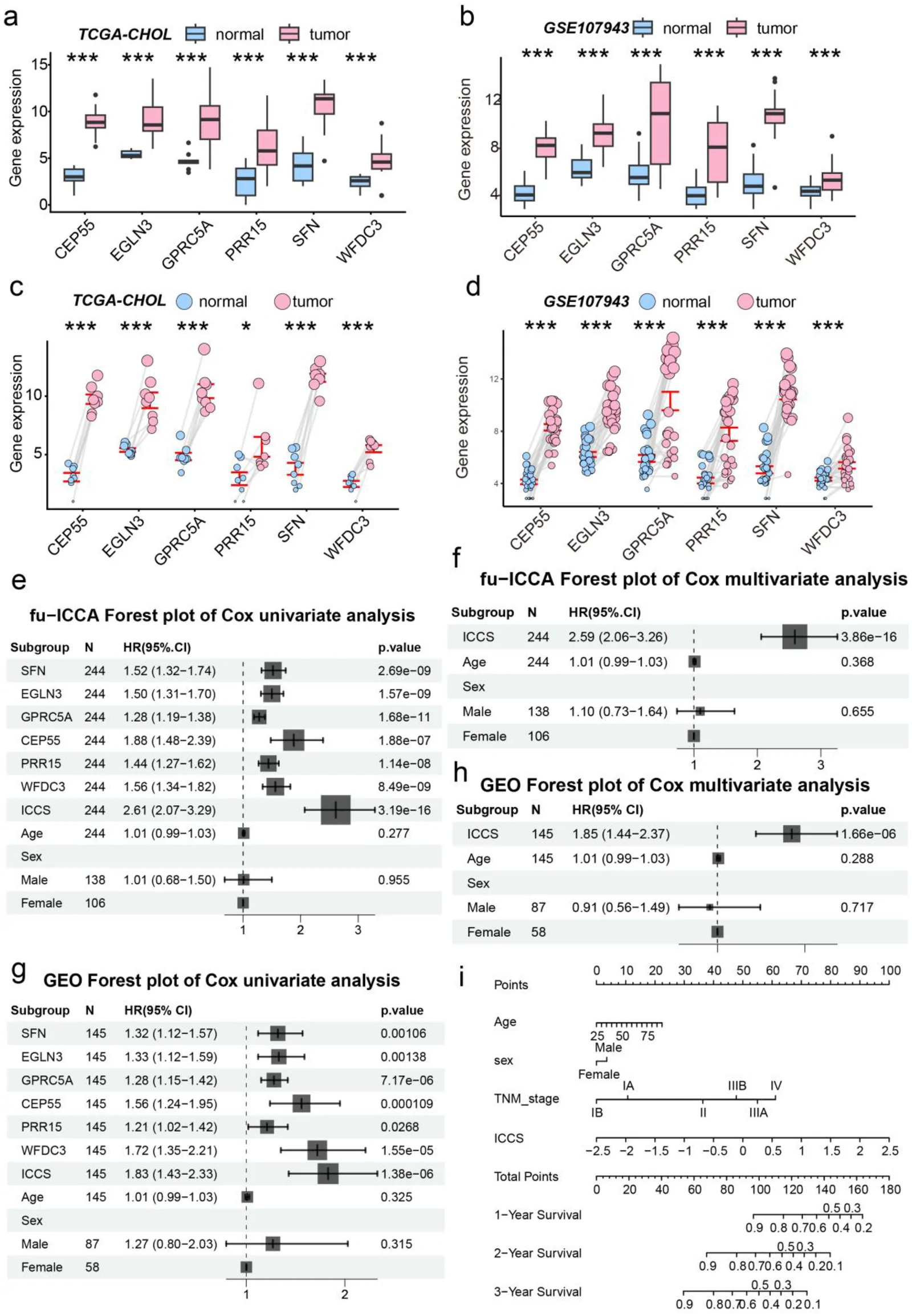
Expression validation, survival analysis, and nomogram construction of core prognostic genes. **(A)** Box plots showing the differential expression of CEP55, EGLN3, GPRC5A, PRR15, SFN, and WFDC3 between tumor and normal tissues in the TCGA cholangiocarcinoma cohort. **(B)** Box plots showing the differential expression of the six ICCS genes between tumor and normal tissues in the GSE107943 cohort. **(C)** Paired comparison of the expression of the six ICCS genes between tumor tissues and matched adjacent normal tissues in the TCGA cholangiocarcinoma cohort. **(D)** Paired comparison of the expression of the six ICCS genes between tumor tissues and matched adjacent normal tissues in the GSE107943 cohort. **(E)** Univariate Cox regression forest plot of the fu-ICCA cohort, showing the association of six genes, ICCS, age, and gender with overall survival. **(F)** Multivariate Cox regression forest plot of the fu-ICCA cohort, including only ICCS, age, and gender clinical variables. **(G)** Univariate Cox regression forest plot of the combined GEO cohort, showing the association of six genes, ICCS, age, and gender with overall survival. **(H)** Multivariate Cox regression forest plot of the combined GEO cohort, including only ICCS, age, and gender clinical variables. **(I)** Nomogram constructed using the fu-ICCA cohort and incorporating ICCS, age, sex, and TNM stage to estimate 1-, 2-, and 3-year overall survival probabilities. Statistical significance is indicated as follows: *P< 0.05, ***P< 0.001.

Based on the variables available in the fu-ICCA cohort, we constructed an exploratory nomogram integrating ICCS, age, and sex to estimate 1-, 2-, and 3-year overall survival probabilities (**Figure 6I**). Because major clinicopathological factors were not comprehensively available for model construction, this nomogram was intended as an exploratory visualization of the prognostic contribution of ICCS rather than as a clinically validated risk-prediction tool. To further assess the potential decision-analytic value of ICCS, we performed decision-curve analysis (DCA) and corresponding net-reduction analysis at selected survival time points (**Supplementary Figure S8**). In the fu-ICCA cohort, ICCS showed positive net benefit over substantial portions of the evaluated threshold-probability range for both 1- and 3-year survival and generally displayed favorable decision-analytic performance relative to the individual ICCS genes and the available clinical variables, including sex, age, and TNM stage (**Supplementary Figures S8a–d**). The corresponding net-reduction analyses similarly suggested that use of ICCS could reduce unnecessary interventions across portions of the evaluated threshold range compared with several individual predictors. In the merged GEO cohort, ICCS also showed favorable net-benefit patterns for 3- and 5-year survival compared with the individual six genes over parts of the evaluated threshold-probability range (**Supplementary Figures S8e, g**). Consistent patterns were observed in the corresponding net-reduction analyses, in which ICCS was associated with greater reductions in unnecessary interventions per 100 patients over selected threshold ranges (**Supplementary Figures S8f, h**). Collectively, these analyses provide exploratory evidence that ICCS may offer additional prognostic decision information beyond individual component genes.

### Computationally inferred immune and metabolic programs and multi-cohort survival associations

To characterize transcriptional programs associated with ICCS stratification, we used the IOBR package to estimate immune- and metabolism-related signature scores in the ICCS-High and ICCS-Low groups (**Figure 7A**). Four signatures were selected for further analysis: Cyclooxygenase_Arachidonic_Acid_Metabolism (CAAM), Poor-Prognosis Angiogenesis Genes (PPAGs), Glycogen_Degradation (GD), and MDSC_peng_et_al, an MDSC-related transcriptional signature. These scores represent computational estimates derived from bulk gene-expression data and should not be interpreted as direct measurements of pathway activation or immune-cell abundance.In the fu-ICCA cohort, higher scores for the CAAM, PPAGs, GD, and MDSC-related signatures were each associated with shorter overall survival (**Figures 7B–E**). These associations were further evaluated in the combined GEO cohort. Higher CAAM, PPAGs, GD, and MDSC-related signature scores were similarly associated with shorter overall survival, with P values of< 0.001, 0.005, 0.004, and< 0.001, respectively (**Figures 7F–I**). These results indicate that higher transcriptome-derived scores for the four immune- and metabolism-related programs were consistently associated with poorer prognosis across the analyzed cohorts.

**Figure 7:**
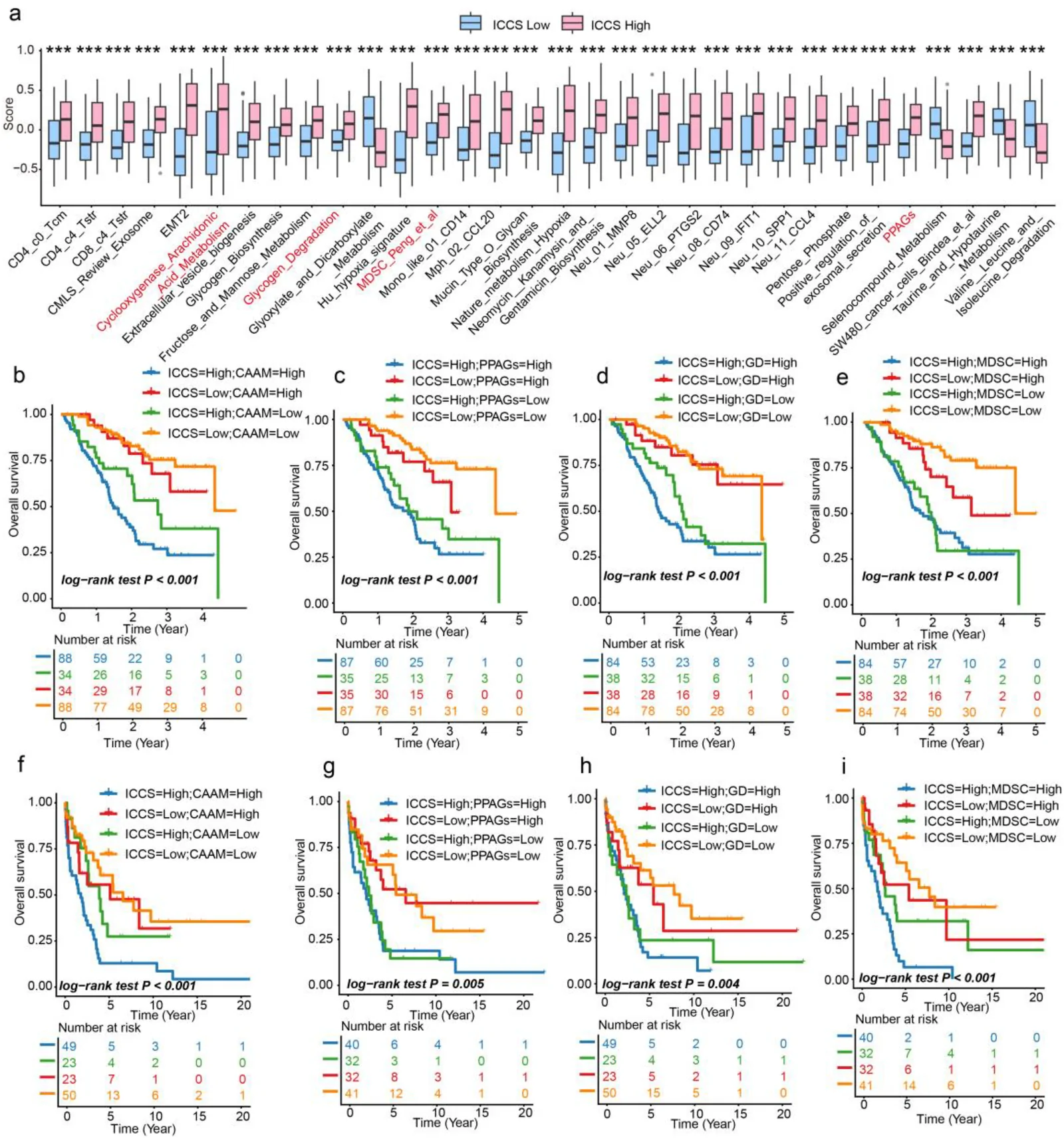
Transcriptome-derived immune and metabolic signatures associated with ICCS and survival. **(A)**Heatmap of pathway enrichment analysis generated using the IOBR R package, showing the activity differences in immune and metabolism-related pathways between the ICCS-High and ICCS-Low subgroups, with four key pathways highlighted. **(B)** Kaplan-Meier survival curves based on CAAM pathway activity stratification in the fu-ICCA cohort. **(C)** Kaplan-Meier survival curves based on PPAGs pathway activity stratification in the fu-ICCA cohort. **(D)** Kaplan-Meier survival curves based on GD pathway activity stratification in the fu-ICCA cohort. **(E)** Kaplan-Meier survival curves based on MDSC pathway activity stratification in the fu-ICCA cohort. **(F)** Kaplan-Meier survival curves based on CAAM pathway activity stratification in the combined GEO cohort. **(G)** Kaplan-Meier survival curves based on PPAGs pathway activity stratification in the combined GEO cohort. **(H)** Kaplan-Meier survival curves based on GD pathway activity stratification in the combined GEO cohort. **(I)** Kaplan-Meier survival curves based on MDSC pathway activity stratification in the combined GEO cohort. Statistical significance is indicated as follows: ***P< 0.001.

### Core gene visualization and WFDC3 regulatory pathway

Based on four spatial transcriptomics samples (cHC-1N, cHC-1L, cHC-1T, ICC-1L), the spatial expression characteristics of six core genes (CEP55, EGLN3, PRR15, SFN, WFDC3, GPRC5A) and the ICCS model score are displayed. All six genes exhibited high expression in tumor tissue (cHC-1T, ICC-1L) and in tumor-margin tissue (cHC-1L), and low expression in normal tissue (cHC-1N). The ICCS model score showed a spatial distribution consistent with these gene expression patterns, with higher scores observed in tumor-associated regions than in the normal section, providing exploratory spatial support for the tumor-associated expression pattern of ICCS(**Figure 8A**). Differential analysis across the four spatial transcriptomics datasets revealed significant expression differences for these six genes (**Figure 8B**), suggesting they may have functional roles in the TME. At the single-cell level, ICCS scores were significantly higher in Scissor+ cells than in Scissor− cells (**Figure 8C**), further supporting the association between higher ICCS scores and prognosis-associated malignant epithelial states. To identify secreted factors, we intersected the six candidate genes with the set of secreted-protein genes in the GeneCards database and obtained two genes: SFN and WFDC3 (**Figure 8D**). Survival analysis of follow-up data showed that WFDC3 had the highest hazard ratio (HR) in both the fu-ICCA and GEO datasets (**Figure 8E**), indicating that WFDC3 may be a key secreted factor influencing patient prognosis. We then used the scTenifoldKnk tool to perform a virtual knockout of WFDC3 in malignant epithelial cells, which produced significant perturbations in the regulation of multiple downstream genes (**Figure 8F**). KEGG functional enrichment analysis showed that genes affected by WFDC3 knockout were mainly involved in pathways such as Cornified envelope formation, Chemical carcinogenesis-DNA adducts, Steroid hormone biosynthesis, the p53 signaling pathway, tight junctions, and glutathione metabolism (**Figure 8G**). Together, these multi-level analyses suggested that WFDC3 may be associated with ICC-related cellular programs and tumor-microenvironment regulation.

**Figure 8:**
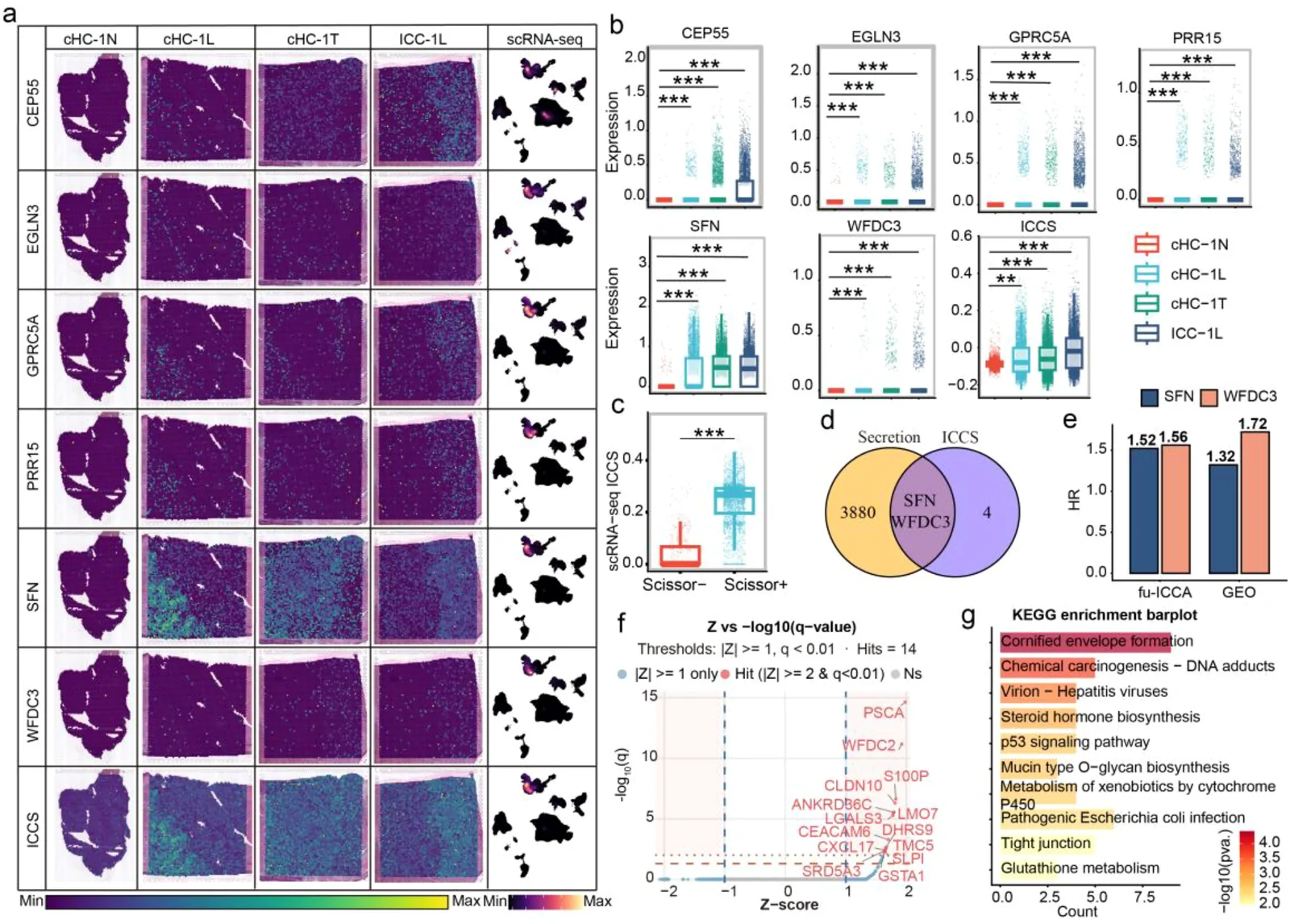
Analysis of six candidate genes and ICCS scores in primary liver cancer and cholangiocarcinoma datasets. **(A)**Spatial and single-cell distribution of six candidate genes (CEP55, EGLN3, GPRC5A, PRR15, SFN, WFDC3) across multiple liver cancer datasets. For each gene, the left panel shows spatial transcriptomics data from four cohorts: cHC-1N, cHC-1L, cHC-1T, and ICC-1L, while the rightmost panel shows scRNA-seq expression patterns. The color scale represents expression levels from minimum (purple) to maximum (yellow). ICCS scores were applied to assess model activity at single-cell resolution. **(B)** Box plots showing differential expression of the six candidate genes and ICCS scores across the four spatial datasets. Each box plot represents the expression distribution of a gene in one cohort. Spatial spot-level distributions are shown descriptively and do not represent independent patient-level biological replicates. **(C)** Comparison of single-cell ICCS scores between Scissor negative (Scissor^−^) and Scissor positive (Scissor^+^) cells. **(D)** Venn diagram showing the overlap of ICCS-associated genes with secreted protein genes from GeneCards, identifying two candidate genes SFN and WFDC3. **(E)** Hazard ratio (HR) analysis of the two overlapping genes in the fu-ICCA and GEO datasets. **(F)** In virtual knockout (KO) of WFDC3 in malignant epithelial cells using scTenifoldKnk, showing its perturbation effect on downstream gene regulatory networks. **(G)** KEGG pathway enrichment analysis of genes with a fold change greater than 1 after in virtual knockout of the WFDC3 gene, highlighting the affected pathways. Statistical significance is indicated as follows: **P< 0.01, ***P< 0.001.

### Mesenchymal–epithelial COL4A1–CD44 signaling in ICCS-high

To define the cellular ecosystem associated with ICCS stratification, tumor-derived single cells were grouped into ICCS-Low and ICCS-High states and visualized across the 11 major cell populations (**Figure 9A**). Marked differences in cellular composition were observed between the two states (**Figure 9B**). In ICCS-Low, T/NK cells represented the largest population (44.28%), whereas epithelial cells accounted for 15.47%. In contrast, ICCS-High was dominated by epithelial cells (78.44%), accompanied by a pronounced reduction in T/NK cells to 3.95%. Mesenchymal cells accounted for 9.75% of ICCS-Low cells and 7.21% of ICCS-High cells. These findings indicate that the high-risk state is characterized by an epithelial-dominant and lymphoid-depleted cellular composition. We next compared inferred intercellular communication between the two ICCS states. Global interaction heatmaps showed that mesenchymal cells were prominent signaling sources in both groups, while mesenchymal-to-epithelial communication was particularly conspicuous in ICCS-High (**Figures 9C, D**). Focused analysis of mesenchymal-to-epithelial signaling identified a broad set of collagen-related ligand–receptor interactions involving CD44, syndecans, and integrin complexes (**Figure 9E**). Contribution analysis ranked COL4A1–CD44 as the leading interaction, followed by COL4A2–CD44 and other collagen–CD44 or collagen–integrin pairs (**Figure 9F**), suggesting that ECM-associated collagen signaling constitutes a major component of mesenchymal–epithelial communication.

**Figure 9:**
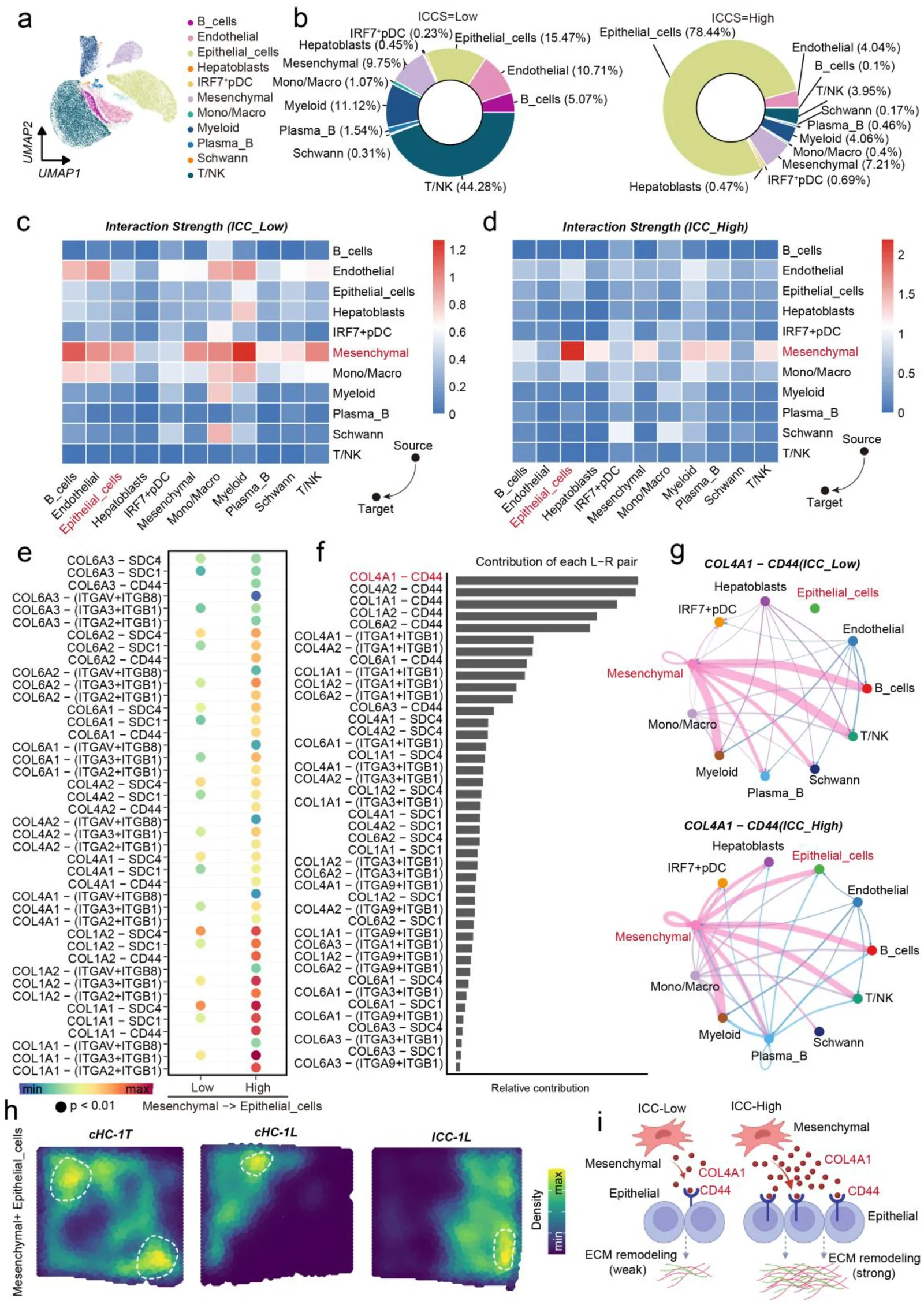
Inferred mesenchymal-to-epithelial collagen signaling and COL4A1–CD44 communication in ICCS-High. **(A)** UMAP visualization of the 11 major cell populations included in the ICCS-stratified single-cell analysis. **(B)** Cellular composition of ICCS-Low and ICCS-High states, shown as donut charts with the proportion of each cell population. **(C, D)** Heatmaps showing inferred cell–cell interaction strength in ICCS-Low **(C)** and ICCS-High **(D)**. Rows represent source cell populations and columns represent target populations; mesenchymal cells and epithelial cells are highlighted. **(E)** Dot plot of significant mesenchymal-to-epithelial ligand–receptor interactions in ICCS-Low and ICCS-High, highlighting collagen-related interactions involving CD44, syndecans, and integrin receptor complexes (p< 0.01). **(F)** Relative contribution of individual ligand–receptor pairs to mesenchymal-to-epithelial communication, with COL4A1–CD44 showing the highest contribution. **(G)** Cell-type communication networks for COL4A1–CD44 in ICCS-Low (top) and ICCS-High (bottom). Edge width represents the relative inferred communication strength. **(H)** Exploratory spatial density maps showing the joint distribution of mesenchymal and epithelial-cell signals in cHC-1T, cHC-1L, and ICC-1L sections. Dashed circles indicate representative regions of local co-density. **(I)** Schematic working model illustrating relatively weak mesenchymal COL4A1–epithelial CD44 signaling and ECM remodeling in ICCS-Low and enhanced COL4A1–CD44-associated signaling with stronger ECM remodeling in ICCS-High. The schematic represents a hypothesis derived from computational and spatial analyses rather than direct mechanistic validation.

Given its highest relative contribution, we further examined the cell-type-level communication pattern of COL4A1–CD44. Network visualization showed a more prominent inferred COL4A1–CD44 communication pattern involving mesenchymal and epithelial cells in the ICCS-High state than in ICCS-Low (**Figure 9G**). Exploratory spatial density maps further demonstrated local regions in which mesenchymal and epithelial signals co-occurred in the cHC-1T, cHC-1L, and ICC-1L sections (**Figure 9H**). Taken together, these analyses support a working model in which the ICCS-High state is accompanied by enhanced mesenchymal collagen signaling toward epithelial CD44 and an ECM-associated microenvironment consistent with stronger matrix remodeling (**Figure 9I**). This interpretation remains hypothesis-generating because the computational analyses do not establish direct ligand–receptor binding or causal ECM remodeling.

### qRT-PCR validation of ICCS genes and functional effects of WFDC3 knockdown

qRT-PCR analysis showed that the mRNA expression levels of CEP55, EGLN3, GPRC5A, PRR15, SFN, and WFDC3 were significantly higher in tumor samples than in normal controls (**Figure 10A**), consistent with the transcriptomic expression patterns observed in the analyzed cohorts. To strengthen the functional evaluation of WFDC3, three independent shRNA sequences targeting WFDC3 (sh-1, sh-2, and sh-3) were initially screened by Western blotting. Among these sequences, sh-1 produced the most pronounced reduction in WFDC3 protein expression and was therefore selected for subsequent functional experiments and designated sh-WFDC3 (**Figure 10B**). WFDC3 knockdown was then independently confirmed in two ICC cell lines, HCCC-9810 and RBE, with Western blotting showing significantly reduced WFDC3 protein expression in sh-WFDC3 cells compared with negative-control (NC) cells (**Figures 10C–F**).

**Figure 10:**
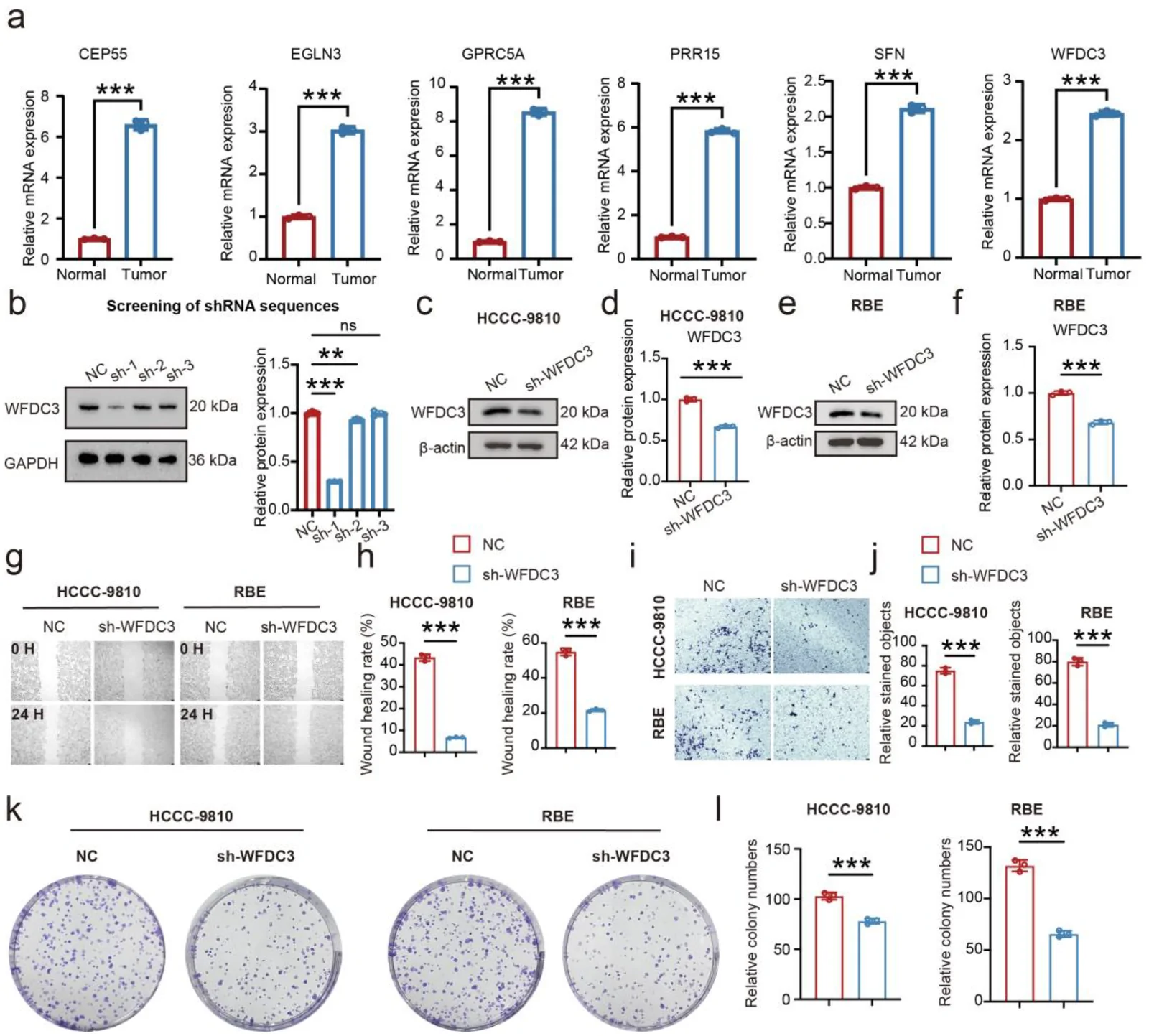
qRT-PCR validation of ICCS genes and functional effects of WFDC3 knockdown in two ICC cell lines. **(A)** Relative mRNA expression levels of CEP55, EGLN3, GPRC5A, PRR15, SFN, and WFDC3 in normal-control and tumor samples, as determined by qRT-PCR. **(B)** Screening of three independent shRNA sequences targeting WFDC3 (sh-1, sh-2, and sh-3) by Western blotting and corresponding densitometric quantification. GAPDH was used as the loading control. sh-1 showed the strongest knockdown efficiency and was selected for subsequent experiments, in which it was designated sh-WFDC3. **(C, D)** Representative Western blot and quantitative analysis of WFDC3 protein expression in NC and sh-WFDC3 HCCC-9810 cells. β-actin was used as the loading control. **(E, F)** Representative Western blot and quantitative analysis of WFDC3 protein expression in NC and sh-WFDC3 RBE cells. **(G)** Representative wound-healing images of NC and sh-WFDC3 HCCC-9810 and RBE cells at 0 and 24 h. **(H)** Quantification of wound-healing rates in HCCC-9810 and RBE cells. **(I)** Representative Transwell invasion images of NC and sh-WFDC3 HCCC-9810 and RBE cells. **(J)** Quantification of Transwell invasion in the two cell lines. **(K)** Representative colony-formation assays in NC and sh-WFDC3 HCCC-9810 and RBE cells. **(L)** Quantification of relative colony numbers in the two cell lines. Quantitative data are presented as mean ± SD from three independent biological experiments. **P< 0.01, ***P< 0.001, ns, not significant.

Functional assays were subsequently performed in both HCCC-9810 and RBE cells. WFDC3 knockdown significantly reduced wound-healing rates in both cell lines, indicating impaired migratory capacity (**Figures 10G, H**). Consistently, Transwell assays showed a marked reduction in the number of invaded cells following WFDC3 knockdown in both HCCC-9810 and RBE cells (**Figures 10I, J**). Colony-formation assays further demonstrated that WFDC3 knockdown significantly decreased clonogenic capacity in both ICC cell lines (**Figures 10K, L**).Taken together, the concordant phenotypic effects observed in HCCC-9810 and RBE cells support the involvement of WFDC3 in the clonogenic, migratory, and invasive phenotypes of ICC cells.

## Discussion

This study integrated single-cell RNA sequencing, spatial transcriptomics, bulk transcriptomics, and machine learning to characterize ICC heterogeneity across multiple analytical levels. We identified prognosis-associated T- and B-cell transcriptional programs, developed a six-gene ICCS signature with reproducible prognostic associations across the analyzed public cohorts, and characterized an ICCS-High state marked by epithelial predominance, reduced T/NK-cell representation, and stronger inferred mesenchymal-to-epithelial collagen signaling. WFDC3 emerged as a prognostically relevant candidate, and its knockdown reduced clonogenic, migratory, and invasive phenotypes in ICC cells. Together, these findings link cellular composition, spatial microenvironmental features, and prognostic heterogeneity in ICC, while their clinical and mechanistic implications require further validation.

T-cell analysis revealed substantial immune-state heterogeneity within ICC. CD8+ T-cell transcriptional modules M1 and M7 were significantly associated with patient prognosis, while pseudotime analysis identified a branched differentiation trajectory in which one branch was enriched for energy-metabolism programs and the other for stress-response pathways. These findings are consistent with the possibility that metabolic and microenvironmental pressures contribute to T-cell dysfunction and regulatory-state transitions within the tumor microenvironment (22, 23). In parallel, ICC samples showed an increased proportion of CD4+ regulatory T cells and a reduced proportion of effector CD8+ T cells. Previous studies have also implicated immunosuppressive mediators such as IL-10 in promoting terminal CD8+ T-cell dysfunction, providing biological context for the T-cell states observed in our analysis (24). B-cell analysis further identified five functional subpopulations, and lower plasma-cell-associated signatures were associated with shorter survival, suggesting that distinct B-cell states may also contribute to disease heterogeneity (25). Taken together, these observations suggest that alterations in both T- and B-cell states may jointly shape the immune microenvironment of ICC (26, 27).

The ICCS prognostic model showed reproducible associations with overall survival across the analyzed public cohorts and remained prognostically informative after adjustment for the available clinical variables. However, these findings represent retrospective evidence derived from publicly available datasets and should not be interpreted as establishing clinical utility (19). At the single-cell level, the ICCS-High state was characterized by greater epithelial-cell representation and markedly reduced T/NK-cell representation, suggesting a distinct immune and cellular composition associated with high-risk disease (28). In parallel, higher CAAM and MDSC-related transcriptional signature scores were associated with ICCS-High and poorer survival, raising the possibility that altered arachidonic acid metabolism and myeloid-related immune programs may contribute to the biological context of this high-risk state (29). Previous studies have shown that cyclooxygenase-dependent arachidonic acid metabolism can promote an immunosuppressive tumor microenvironment, in part through enhanced recruitment or accumulation of myeloid-derived suppressor cells and consequent impairment of T-cell function (30, 31). Together with these previous observations, our transcriptomic findings support further investigation of arachidonic acid metabolism as a potential component of ICC-associated immune regulation, rather than establishing a direct causal mechanism (32, 33).

Several limitations should be acknowledged. First, the single-cell, spatial transcriptomic, and bulk transcriptomic datasets used for discovery, model development, and prognostic evaluation were derived primarily from publicly available resources or previously published cohorts. Although additional qRT-PCR and functional experiments were performed in the present study, these experiments were designed to validate gene-expression patterns and the cellular function of WFDC3 and did not constitute an independent clinical cohort for prognostic validation of ICCS. Therefore, the generalizability and clinical utility of ICCS remain to be established in independent institutional cohorts with comprehensive clinicopathological information and longitudinal follow-up. In addition, the present study did not develop or analytically validate a clinically deployable assay for the six-gene ICCS signature. The qRT-PCR experiment was performed as expression-level validation rather than as a diagnostic or prognostic test, and no standardized clinical workflow, predefined clinical cutoff, inter-assay reproducibility assessment, or IHC/qPCR-based implementation strategy was established. Second, heterogeneity among the public datasets represents an additional source of uncertainty. Differences in sequencing platforms, sample processing, normalization procedures, patient populations, disease characteristics, treatment histories, and follow-up may introduce technical batch effects and cohort-specific biological variation. Such heterogeneity may influence absolute gene-expression levels, estimated cellular composition, ICCS score distributions, and the observed discrimination and calibration of the prognostic model. Although cross-dataset integration and validation were used to reduce the influence of dataset-specific effects, residual technical and biological heterogeneity cannot be completely excluded. Third, the spatial transcriptomic analysis was based on only four sections with heterogeneous histological origins. Accordingly, the observed spatial niches, gene-expression patterns, and mesenchymal–epithelial co-localization should be regarded as section-level, hypothesis-generating observations rather than population-level evidence. Fourth, collagen-related ligand–receptor interactions, including COL4A1–CD44, were computationally inferred and do not establish direct molecular binding, downstream signaling activation, or causal ECM remodeling. Spatial co-density similarly indicates local co-occurrence rather than direct cell–cell communication. Future studies should evaluate ICCS in independent institutional and multicenter cohorts using standardized expression assays and prespecified risk thresholds, followed by prospective validation with comprehensive clinicopathological adjustment. Larger spatial cohorts and protein-level spatial validation will also be required to determine the reproducibility and biological relevance of the identified stromal–epithelial patterns.

In summary, integrative multi-omics analysis identified a six-gene ICCS signature and an ICCS-High state characterized by epithelial predominance, reduced T/NK-cell representation, and stronger inferred mesenchymal-to-epithelial collagen signaling. COL4A1–CD44 emerged as a high-contribution candidate interaction, while WFDC3 knockdown supported a role for WFDC3 in malignant ICC cell phenotypes. These findings provide a framework for investigating prognostic heterogeneity in ICC. ICCS remains a research-stage signature that requires standardized assays and independent prospective validation before clinical application.

## Data Availability

The original contributions presented in the study are included in the article/**Supplementary Material**. Further inquiries can be directed to the corresponding authors.
