# Supplementary material for: Single-cell and spatial multi-omics combined with machine learning to unravel the heterogeneity of cholangiocarcinoma and the WFDC3-mediated prognostic regulatory mechanisms
Source: Front Immunol. 2026 Oct 8;17:1903609. doi: 10.3389/fimmu.2026.1903609 (PMC13687428; doi:10.3389/fimmu.2026.1903609)
Supplement: Supplementary file 1 [file DataSheet1.pdf]

Single-Cell and Spatial Multi-Omics Combined with Machine Learning to Unravel the Heterogeneity of Cholangiocarcinoma and the WFDC3-Mediated Prognostic Regulatory Mechanisms

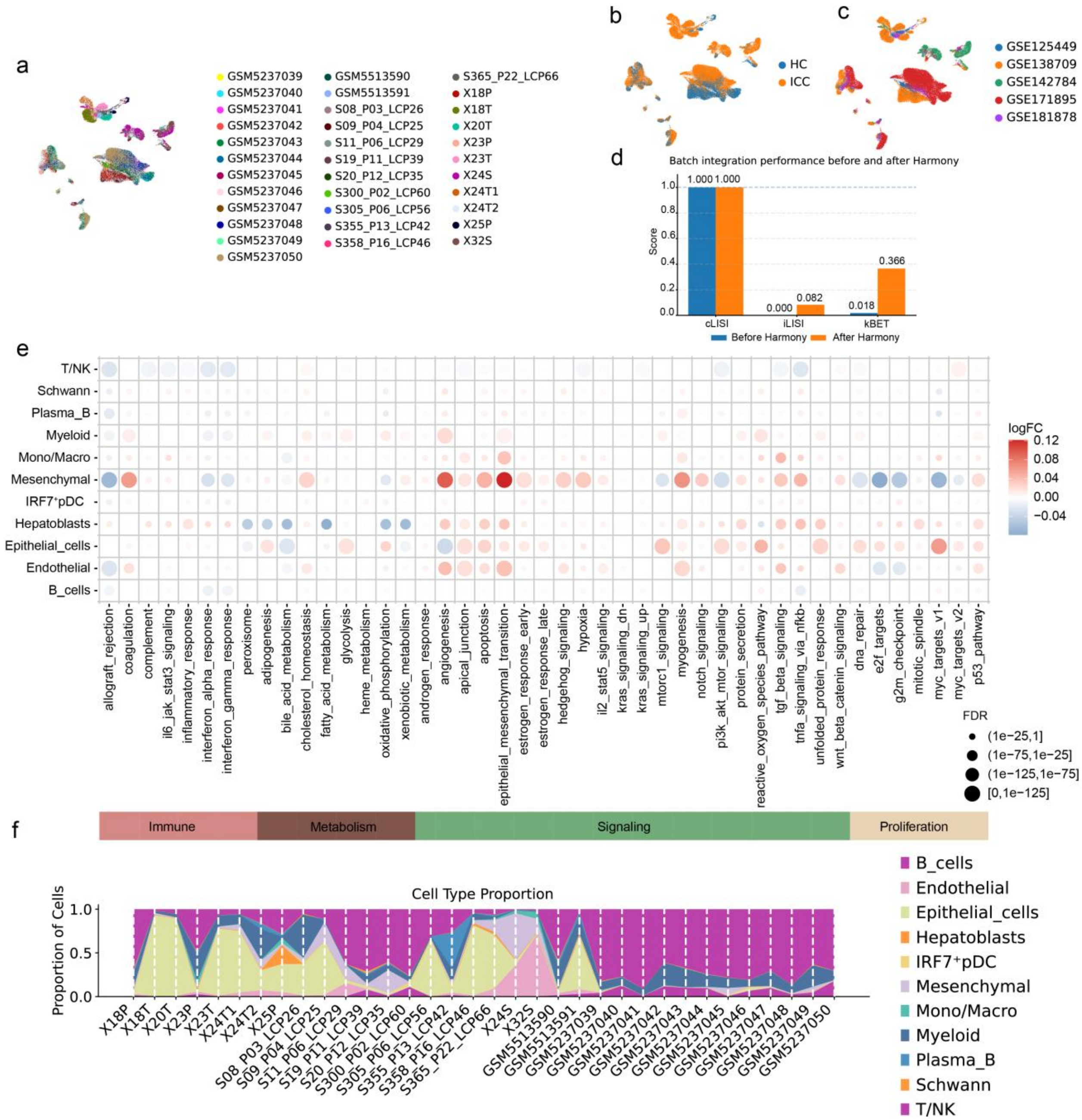

Figure S1. Supplementary evaluation of single-cell transcriptome integration and functional characteristics after clustering.(a) Harmony-corrected UMAP visualization colored by individual sample, showing the distribution of cells from each sample in the integrated single-cell atlas.(b) Harmony-corrected UMAP visualization colored by disease group, including the combined hepatocellular–cholangiocarcinoma control group (cHC) and the intrahepatic cholangiocarcinoma group (ICC).(c) Harmony-corrected UMAP visualization colored by the five GEO dataset sources: GSE125449, GSE138709, GSE142784,

GSE171895, and GSE181878.(d) Quantitative comparison of integration performance before and after Harmony correction using the scaled cell-type local inverse Simpson's index (cLISI), scaled integration local inverse Simpson's index (iLISI), and cell-type-aware k-nearest-neighbor batch-effect test (kBET) acceptance rate. Higher scaled cLISI values indicate better preservation of cell-type identity, whereas higher scaled iLISI and kBET values indicate improved mixing across datasets.(e) Bubble plot showing differences in the activity of cholangiocarcinoma-related pathways across the 11 major cell types. Pathways were categorized into immune, metabolic, signaling, and proliferation-related programs. Bubble color represents the log fold change (logFC), and bubble size represents the false discovery rate (FDR).(f) Sample-level stacked proportion plot showing the relative abundance of the 11 major cell types across individual samples.

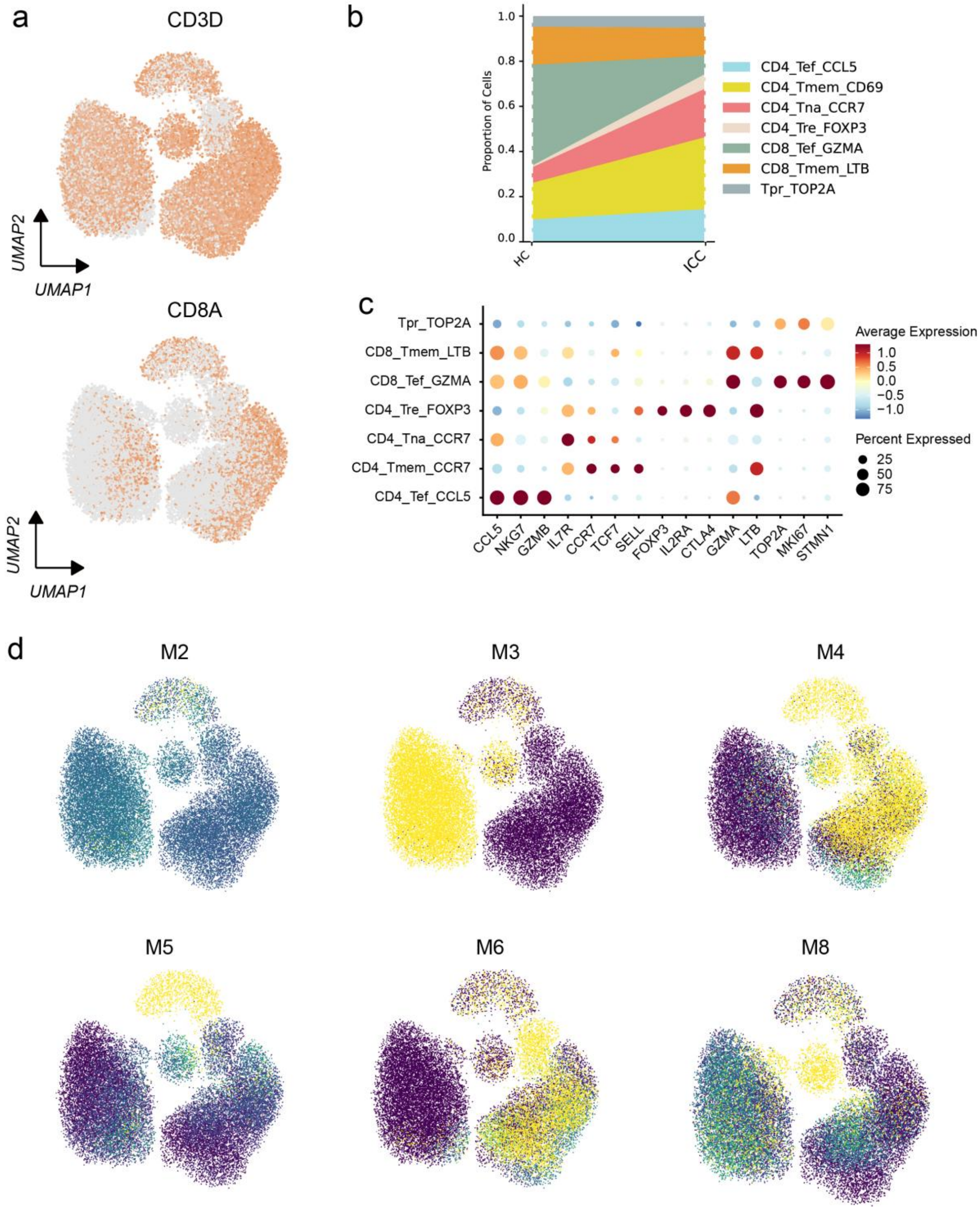

Figure S2. Supplementary Feature Analysis of T Cell Composition and Hotspot Gene Modules

(a) UMAP feature plots showing the expression of the pan-T-cell marker CD3D and the cytotoxic T-cell marker CD8A in the re-clustered T-cell population.(b) Stacked bar plot showing the relative proportions of the seven T-cell subpopulations in healthy control (HC) and intrahepatic cholangiocarcinoma (ICC) samples.(c) Dot plot showing the expression of representative marker genes across the seven T-cell subpopulations. Dot size indicates the percentage of cells expressing each gene, whereas color represents the scaled average expression level.(d) UMAP visualization of representative Hotspot-derived

transcriptional modules, including M2, M3, M4, M5, M6, and M8, in $CD8^+$ T cells. Colors indicate the relative module activity scores across individual cells.

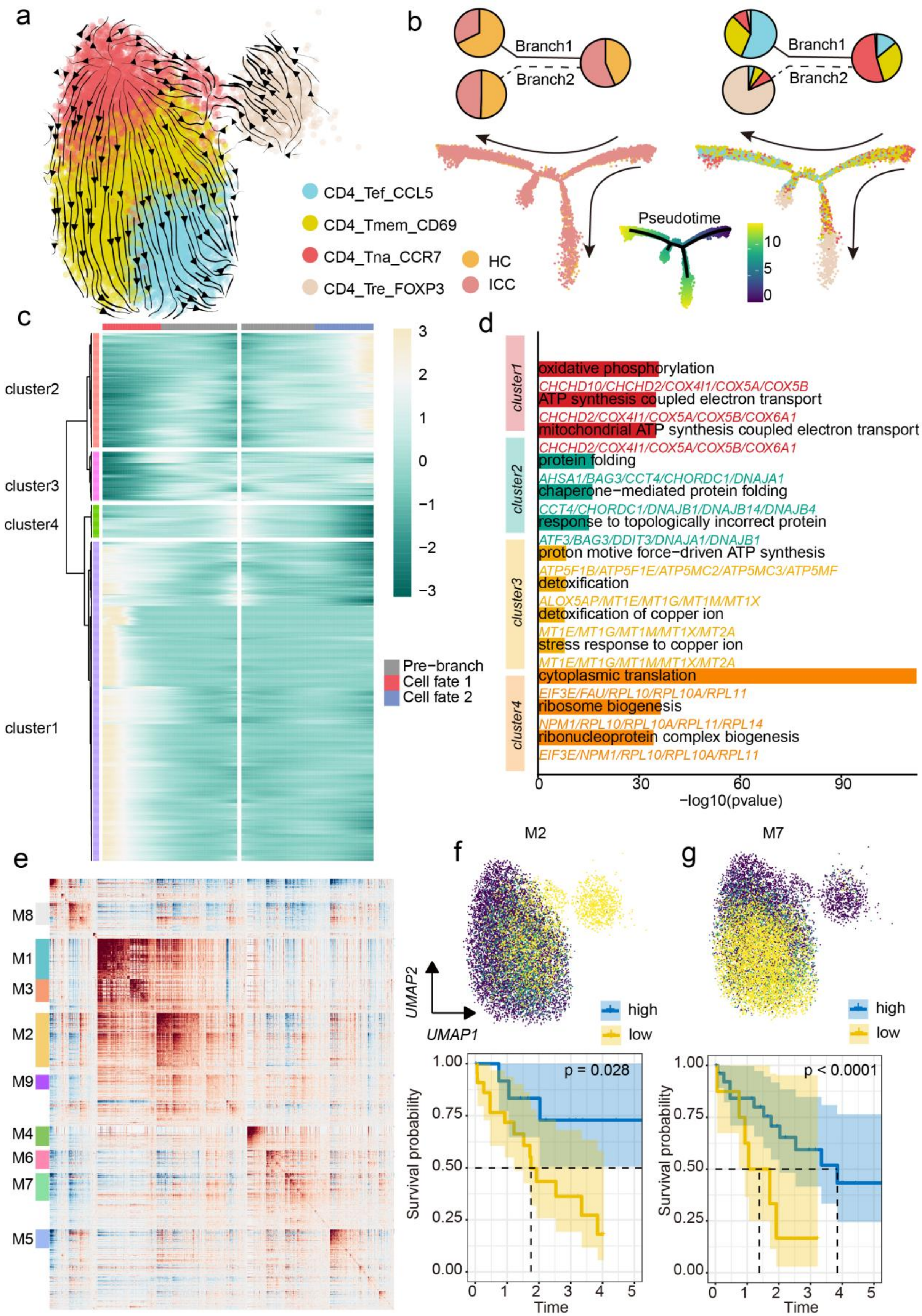

a
CD4_Tef_CCL5
CD4_Tmem_CD69
CD4_Tna_CCR7
CD4_Tre_FOXP3
HC
ICC
b
Branch1
Branch2
Pseudotime
c
cluster2
cluster3
cluster4
cluster1
Pre−branch
Cell fate 1
Cell fate 2
d
cluster1
cluster2
cluster3
cluster4
oxidative phosphorylation
CHCHD10/CHCHD2/COX4I1/COX5A/COX5B
ATP synthesis coupled electron transport
CHCHD2/COX4I1/COX5A/COX5B/COX6A1
mitochondrial ATP synthesis coupled electron transport
CHCHD2/COX4I1/COX5A/COX5B/COX6A1
protein folding
AHSA1/BAG3/CCT4/CHORDC1/DNAJA1
chaperone−mediated protein folding
CCT4/CHORDC1/DNAJB1/DNAJB14/DNAJB4
response to topologically incorrect protein
ATF3/BAG3/DDIT3/DNAJA1/DNAJB1
proton motive force−driven ATP synthesis
ATP5F1B/ATP5F1E/ATP5MC2/ATP5MC3/ATP5MF
detoxification
ALOX5AP/MT1E/MT1G/MT1M/MT1X
detoxification of copper ion
MT1E/MT1G/MT1M/MT1X/MT2A
stress response to copper ion
MT1E/MT1G/MT1M/MT1X/MT2A
cytoplasmic translation
EIF3E/FAU/RPL10/RPL10A/RPL11
ribosome biogenesis
NPM1/RPL10/RPL10A/RPL11/RPL14
ribonucleoprotein complex biogenesis
EIF3E/NPM1/RPL10/RPL10A/RPL11
−log10(pvalue)
e
M8
M1
M3
M2
M9
M4
M6
M7
M5
f
M2
g
M7
UMAP2
UMAP1
high
low
p = 0.028
p < 0.0001
Survival probability
Time

Figure S3. CD4+ T-cell differentiation trajectories and prognostic analysis of Hotspot modules.

(a) CellRank-based trajectory showing the differentiation directions of CD4+ T-cell subpopulations.(b) Monocle-based trajectory showing CD4+ T-cell pseudotime progression, branch structure, and the distribution of HC and ICC cells across Fate1 and Fate2.(c) Heatmap of branch-dependent genes across the pre-branch, Fate1, and Fate2 states.(d) Functional enrichment of gene clusters associated with CD4+ T-cell differentiation trajectories.(e) Heatmap of nine CD4+ T-cell Hotspot modules (M1–M9).(f, g) UMAP distributions and Kaplan–Meier survival curves for CD4+ T-cell modules M2 and M7.

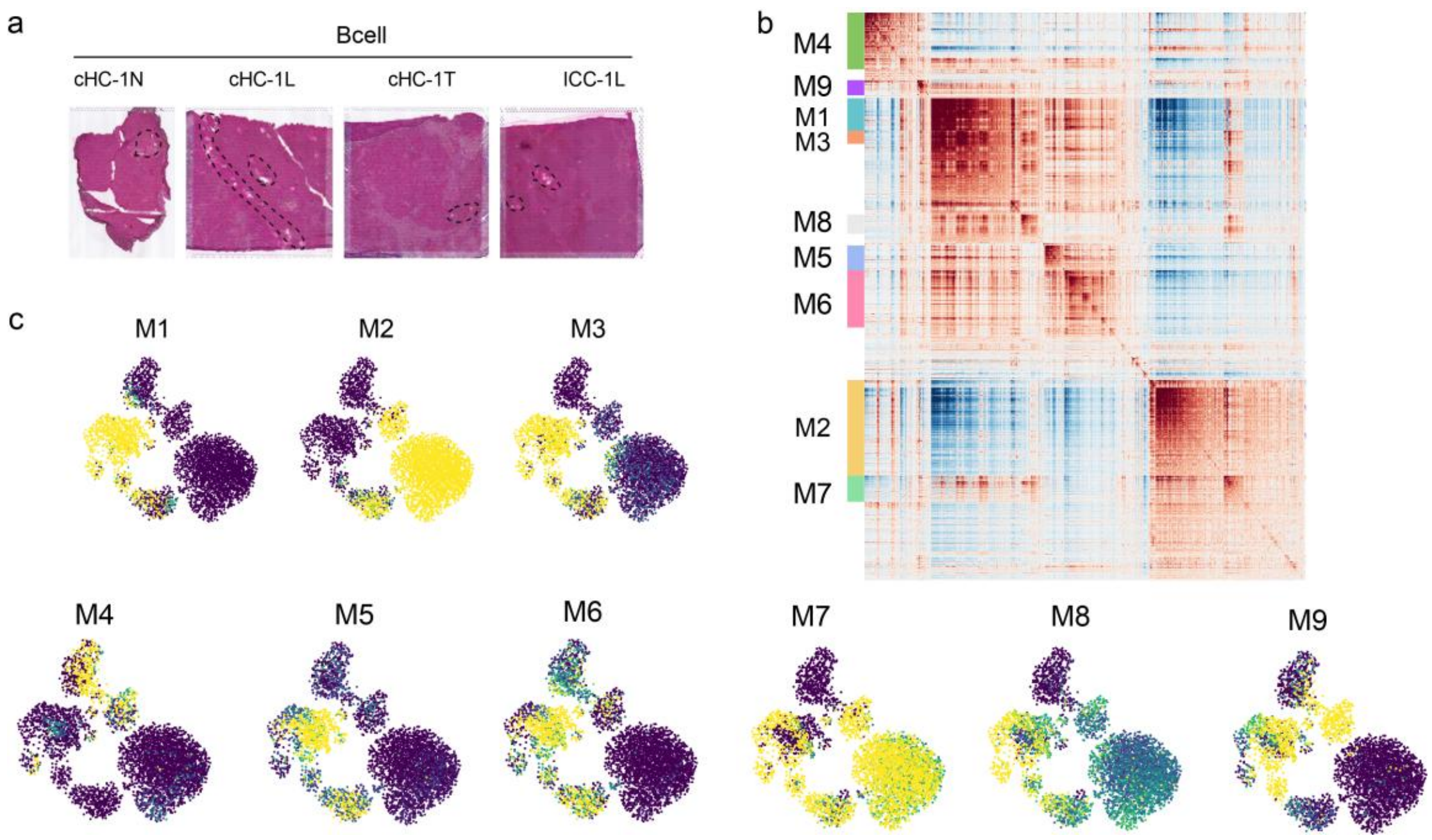

Figure S4. Spatial localization and expression characteristics of cell subgroups

(a) Spatial localization map of B cells in different tissue samples (cHC, ICC), with dashed boxes indicating B cell enrichment regions; (b) Heatmap of expression association between B cell subgroups (M1-M9) and functional genes; (c) UMAP expression distribution map of 9 B cell subgroups.

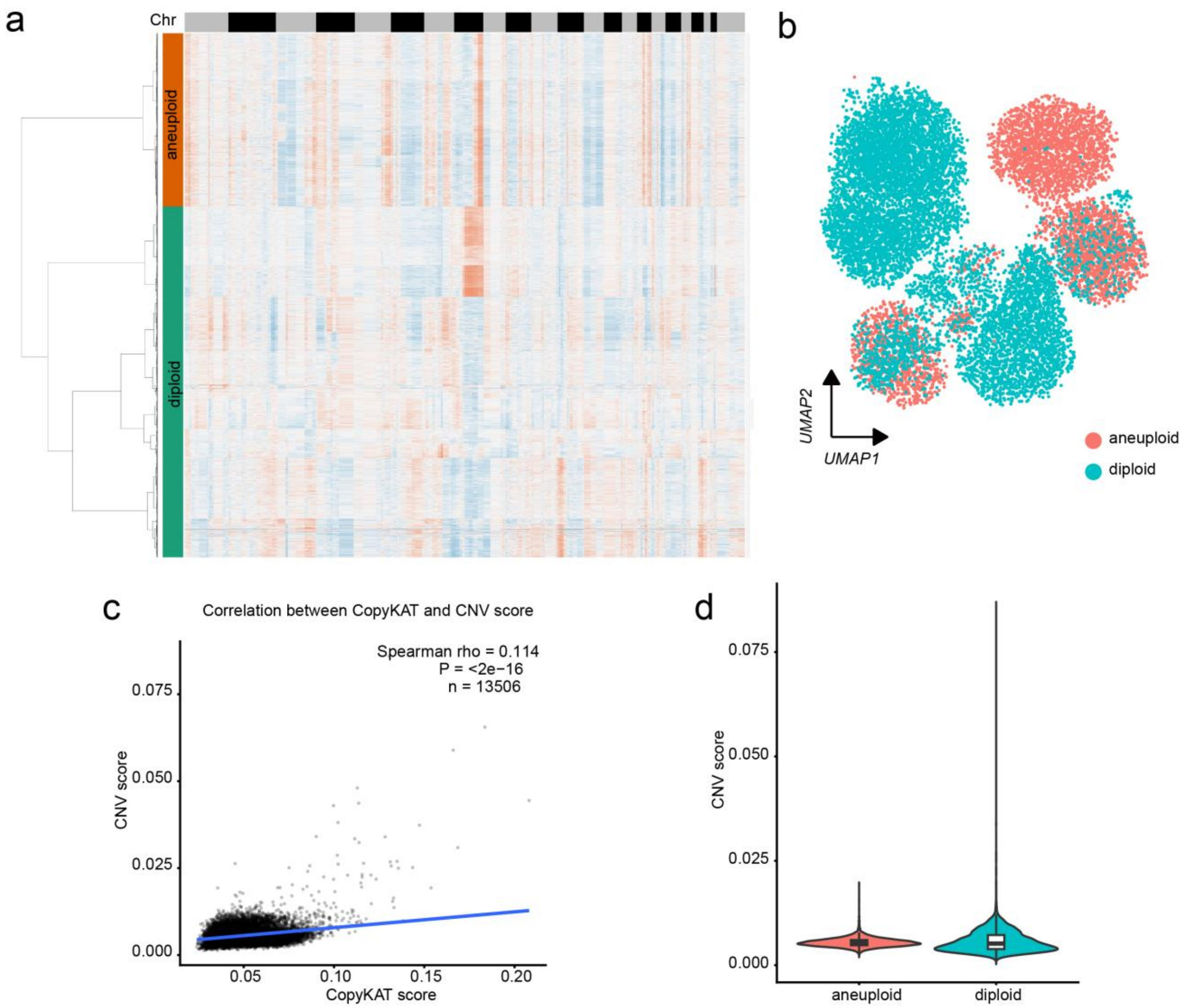

Figure S5. Orthogonal evaluation of InferCNV-derived epithelial copy-number states using CopyKAT.

(a) CopyKAT-derived chromosome-scale copy-number heatmap of epithelial cells. Cells were classified as aneuploid or diploid according to their inferred genome-wide copy-number profiles. Chromosome positions are indicated along the top.(b) UMAP visualization of epithelial cells colored by CopyKAT classification. Red represents aneuploid cells, and turquoise represents diploid cells.(c) Spearman correlation between CopyKAT-derived scores and InferCNV-derived CNV scores across 13,506 epithelial cells. The blue line represents the fitted trend. The Spearman correlation coefficient and p value are shown.(d) Violin plots showing the distributions of InferCNV-derived CNV scores in CopyKAT-defined aneuploid and diploid cells. The embedded box plots indicate the median and interquartile range.

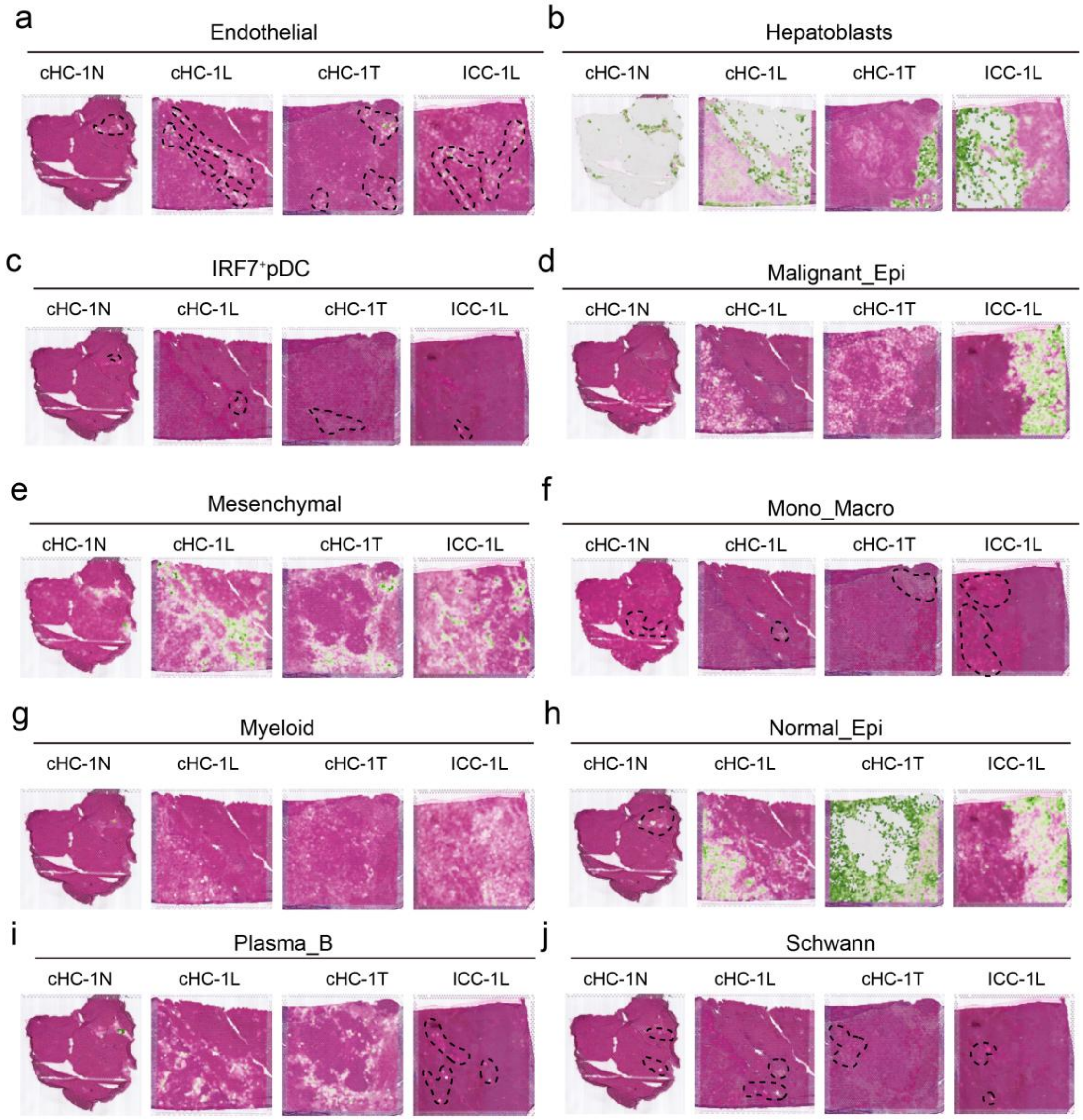

Figure S6. Spatial Distribution of Different Cell Types in Cholangiocarcinoma and Paracancerous Tissues

(a-j) Spatial transcriptomic visualization results of 10 core cell types in cHC and ICC samples. Green areas represent the enrichment locations of the corresponding cells.

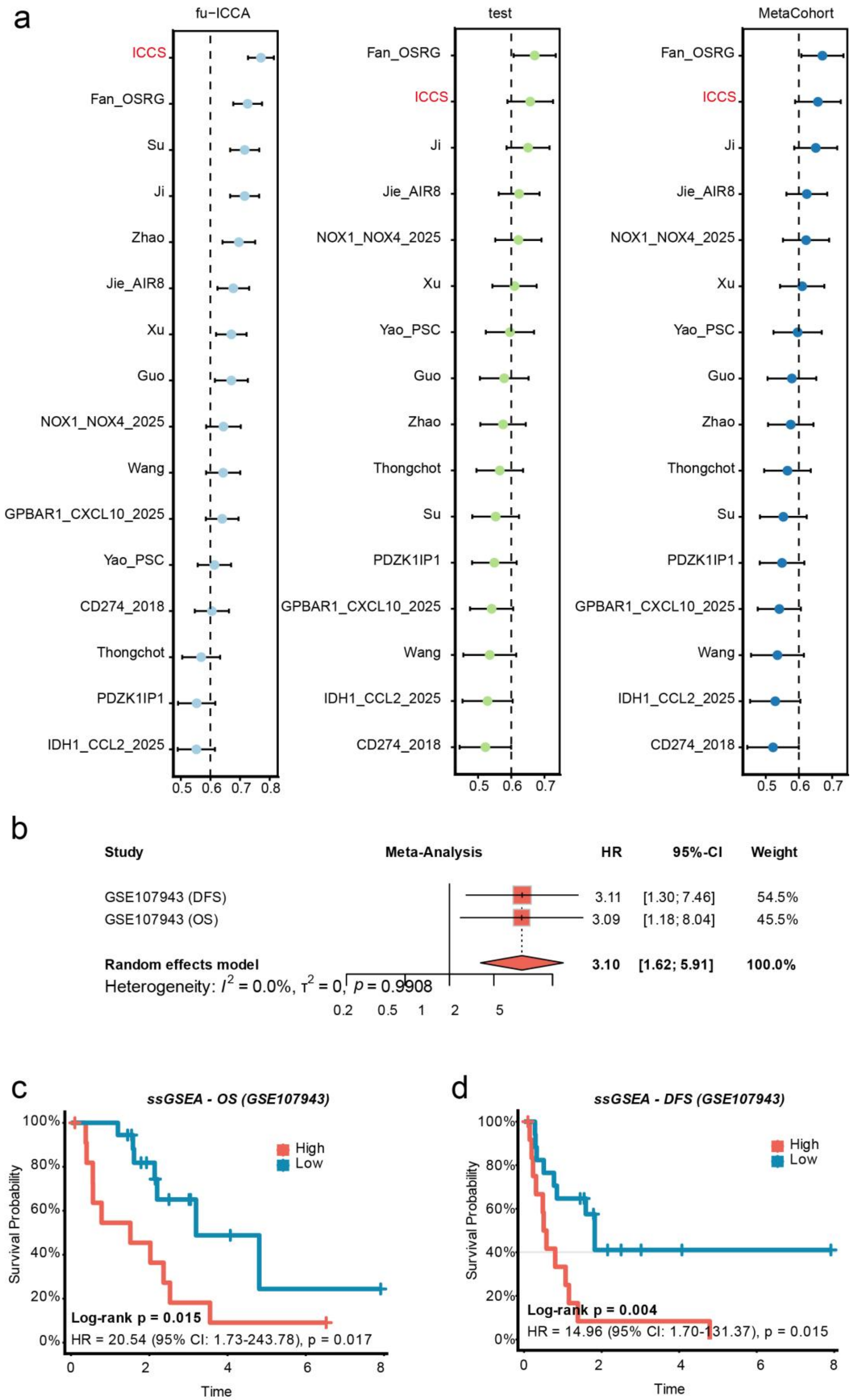

a
fu−ICCA
test
MetaCohort
ICCS
Fan_OSRG
Su
Ji
Zhao
Jie_AIR8
Xu
Guo
NOX1_NOX4_2025
Wang
GPBAR1_CXCL10_2025
Yao_PSC
CD274_2018
Thongchot
PDZK1IP1
IDH1_CCL2_2025
b
Study
Meta-Analysis
HR
95%-CI
Weight
GSE107943 (DFS) 3.11 [1.30; 7.46] 54.5%
GSE107943 (OS) 3.09 [1.18; 8.04] 45.5%
Random effects model 3.10 [1.62; 5.91] 100.0%
Heterogeneity: I^2 = 0.0%, τ^2 = 0, p = 0.9908
0.2 0.5 1 2 5
c
ssGSEA - OS (GSE107943)
High
Low
Survival Probability
Log-rank p = 0.015
HR = 20.54 (95% CI: 1.73-243.78), p = 0.017
Time
d
ssGSEA - DFS (GSE107943)
Log-rank p = 0.004
HR = 14.96 (95% CI: 1.70-131.37), p = 0.015

Figure S7. Comparative and survival evaluation of the ICCS signature.

(a) C-index comparison of ICCS and published ICC prognostic signatures across three cohorts. (b) Associations of ICCS with disease-free survival and overall survival in GSE107943. (c, d) Kaplan–Meier curves for overall survival and disease-free survival, respectively, according to the six-gene ICCS ssGSEA score in GSE107943.

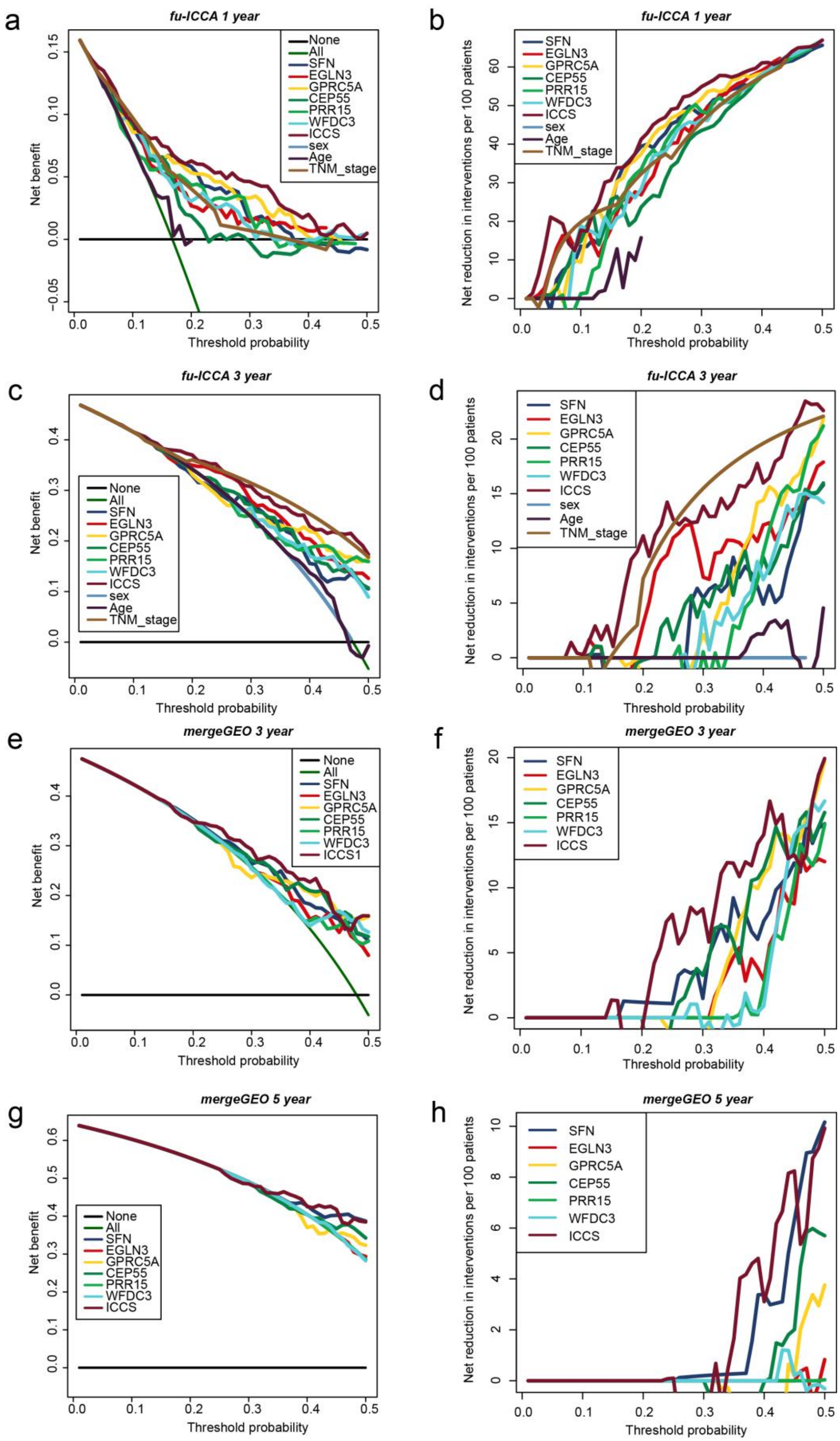
a
fu-ICCA 1 year
None
All
SFN
EGLN3
GPRC5A
CEP55
PRR15
WFDC3
ICCS
sex
Age
TNM_stage
Net benefit
Threshold probability
b
fu-ICCA 1 year
SFN
EGLN3
GPRC5A
CEP55
PRR15
WFDC3
ICCS
sex
Age
TNM_stage
Net reduction in interventions per 100 patients
Threshold probability
c
fu-ICCA 3 year
None
All
SFN
EGLN3
GPRC5A
CEP55
PRR15
WFDC3
ICCS
sex
Age
TNM_stage
Net benefit
Threshold probability
d
fu-ICCA 3 year
SFN
EGLN3
GPRC5A
CEP55
PRR15
WFDC3
ICCS
sex
Age
TNM_stage
Net reduction in interventions per 100 patients
Threshold probability
e
mergeGEO 3 year
None
All
SFN
EGLN3
GPRC5A
CEP55
PRR15
WFDC3
ICCS1
Net benefit
Threshold probability
f
mergeGEO 3 year
SFN
EGLN3
GPRC5A
CEP55
PRR15
WFDC3
ICCS
Net reduction in interventions per 100 patients
Threshold probability
g
mergeGEO 5 year
None
All
SFN
EGLN3
GPRC5A
CEP55
PRR15
WFDC3
ICCS
Net benefit
Threshold probability
h
mergeGEO 5 year
SFN
EGLN3
GPRC5A
CEP55
PRR15
WFDC3
ICCS
Net reduction in interventions per 100 patients
Threshold probability

Figure S8. Exploratory decision-curve and net-reduction analyses of ICCS in the fu-ICC and merged GEO cohorts.

(a) Decision-curve analysis for 1-year survival in the fu-ICC cohort, comparing the net benefit of the six individual ICCS genes (SFN, EGLN3, GPRC5A, CEP55, PRR15, and WFDC3), the composite ICCS score, and the available clinical variables (sex, age, and TNM stage) across a range of threshold probabilities. “All” and “None” indicate the treat-all and treat-none reference strategies, respectively. (b) Corresponding net-reduction curve for 1-year survival, showing the estimated net reduction in interventions per 100 patients across threshold probabilities. (c,d) Decision-curve analysis and corresponding net-reduction analysis for 3-year survival in the fu-ICC cohort. (e,f) Decision-curve analysis and corresponding net-reduction analysis for 3-year survival in the merged GEO cohort, comparing the six ICCS genes with the composite ICCS score. (g,h) Decision-curve analysis and corresponding net-reduction analysis for 5-year survival in the merged GEO cohort. These analyses were performed as exploratory assessments of potential decision-analytic value and do not constitute prospective validation of clinical utility.
